# The Repertoire and Dynamics of Evolutionary Adaptations to Controlled Nutrient-Limited Environments in Yeast

**DOI:** 10.1371/journal.pgen.1000303

**Published:** 2008-12-12

**Authors:** David Gresham, Michael M. Desai, Cheryl M. Tucker, Harry T. Jenq, Dave A. Pai, Alexandra Ward, Christopher G. DeSevo, David Botstein, Maitreya J. Dunham

**Affiliations:** 1 Lewis-Sigler Institute for Integrative Genomics, Princeton University, Princeton, New Jersey, United States of America; 2 Department of Molecular Biology, Princeton University, Princeton, New Jersey, United States of America; 3 Department of Genome Sciences, University of Washington, Seattle, Washington, United States of America; Yale University, United States of America

## Abstract

The experimental evolution of laboratory populations of microbes provides an opportunity to observe the evolutionary dynamics of adaptation in real time. Until very recently, however, such studies have been limited by our inability to systematically find mutations in evolved organisms. We overcome this limitation by using a variety of DNA microarray-based techniques to characterize genetic changes—including point mutations, structural changes, and insertion variation—that resulted from the experimental adaptation of 24 haploid and diploid cultures of *Saccharomyces cerevisiae* to growth in either glucose, sulfate, or phosphate-limited chemostats for ∼200 generations. We identified frequent genomic amplifications and rearrangements as well as novel retrotransposition events associated with adaptation. Global nucleotide variation detection in ten clonal isolates identified 32 point mutations. On the basis of mutation frequencies, we infer that these mutations and the subsequent dynamics of adaptation are determined by the batch phase of growth prior to initiation of the continuous phase in the chemostat. We relate these genotypic changes to phenotypic outcomes, namely global patterns of gene expression, and to increases in fitness by 5–50%. We found that the spectrum of available mutations in glucose- or phosphate-limited environments combined with the batch phase population dynamics early in our experiments allowed several distinct genotypic and phenotypic evolutionary pathways in response to these nutrient limitations. By contrast, sulfate-limited populations were much more constrained in both genotypic and phenotypic outcomes. Thus, the reproducibility of evolution varies with specific selective pressures, reflecting the constraints inherent in the system-level organization of metabolic processes in the cell. We were able to relate some of the observed adaptive mutations (e.g., transporter gene amplifications) to known features of the relevant metabolic pathways, but many of the mutations pointed to genes not previously associated with the relevant physiology. Thus, in addition to answering basic mechanistic questions about evolutionary mechanisms, our work suggests that experimental evolution can also shed light on the function and regulation of individual metabolic pathways.

## Author Summary

Adaptive evolution is a central biological process that underlies diverse phenomena from the acquisition of antibiotic resistance by microbes to the evolution of niche specialization. Two unresolved questions regarding adaptive evolution are what types of genomic variation are associated with adaptation and how repeatable is the process. We evolved yeast populations for more than 200 generations in nutrient-limited chemostats. We find that the phenotype of adapted individuals, as measured using global gene expression, is much less variable in clones adapted to sulfate limitation than either glucose or phosphate limitation. We comprehensively analyzed the genomes of adapted clones and found that those adapted to sulfate limitation almost invariably carry amplifications of the gene encoding a sulfur transporter, but the mutations in individuals adapted to glucose and phosphate limitation are much more diverse. This parallelism holds true at the level of single-nucleotide mutations. Although there may be other paths to adapt to sulfate limitation, one path confers a much greater advantage than all others so it dominates. By contrast, there are a number of ways to adapt to glucose and phosphate limitation that confer similar advantages. We conclude that the reproducibility of evolution depends on the specific selective pressure experienced by the organism.

## Introduction

The study of organismal evolution at the molecular level is a potent means of understanding how genomes evolve in response to selective pressures. Most kinds of evolutionary analysis are necessarily retrospective: individuals are sampled from a population in the present, genetic variation is assessed, and inferences about the past action of evolutionary forces are drawn from the patterns of observed variation. Yet by their nature, retrospective analyses based on variation at a snapshot in time cannot directly address the dynamics of evolution. Experimental evolution of microbes provides an alternative to this retrospective approach: short generation times and the ease of maintaining sizable populations make it feasible to observe adaptation in real time. The study of experimental evolution of microbes in controlled laboratory environments has a long history, beginning with the demonstration by Luria and Delbrück [1] that adaptive mutations exist in populations prior to selection. However, until very recently this approach has suffered from one key limitation: there was no way to detect new mutations genome-wide and to trace their fate through the experiment. Instead, analysis has focused primarily on interpreting phenotypic outcomes (e.g. increases in fitness and other acquired phenotypes), without being able to make a direct connection to the genetic variability underlying these phenotypes. Thus, many basic questions regarding evolutionary mechanisms have yet to be successfully addressed experimentally.

First, we do not know how many mutations we expect cells to accumulate in a given time in a given environment, nor what fraction of these mutations will be neutral or contribute to large or small increases in fitness, although a few recent studies in phage [2],[3] and bacteria [4],[5] have begun to address these questions. Fundamentally this is a question of what mutation rates are, what the distribution of selective effects of new mutations is, how these vary with organism and environment, and how these parameters interact with particular population dynamics to determine how evolution progresses. Experimental measurements of these quantities and theoretical understanding of how they interact have both proven very challenging, though some progress has been made [6]–[10]. Second, we do not know what variability in any of these quantities to expect within or between populations. Will evolution in the same controlled environment carried out many times result in fitter variants with identical, similar, or highly divergent sets of mutations? How will this genotypic diversity relate to diversity in physiology? More specifically, for any given nutrient limitation, how severely does the system-level organization of metabolism limit the evolutionary possibilities? While extensive experimental work has measured both genetic and phenotypic variability (e.g. [11],[12]), systematic analysis of the relationship between genome-wide genetic variation and a quantifiable measure of phenotypic variation has not been done, nor has this been connected to the organization of metabolic pathways. Finally, we have a relatively limited understanding of the nature of the mutational spectrum on which evolution operates. Specifically, we do not have much experimental information about the relative importance of point mutations, transposon insertions, or genome rearrangements in evolution.

Microarray-based genomic technologies provide tools to tackle some of these basic questions, by allowing us to systematically find most mutations genome-wide in evolved strains, and track their fates through the experiments [5], [13]–[16]. These methods allow global characterization of the genomes of clonal isolates recovered from populations evolved under experimental conditions, including structural and insertional genomic variation as well as point mutations [16], though they still may miss important classes of mutations in repeats and other low complexity sequences. Recent analyses of genomes of experimentally evolved yeast [14] and bacterial strains [5] have demonstrated that the number of mutations associated with long-term experimental evolutions is small, making the task of complete characterization of mutational spectra in adapted organisms feasible. Thus, microarray approaches to characterizing genomes have the potential to facilitate determination of the causation of complex phenotypic outcomes of experimental evolution experiments such as global transcriptional [17], translational [18] and metabolic [19] states.

In this paper we begin to address the above questions by following evolutionary adaptation of cultures of the single-celled eukaryote, *Saccharomyces cerevisiae*, growing at steady state under three different metabolic limitations, and assessing the extent and dynamics of genotypic and phenotypic diversity. We subjected 24 yeast populations to ∼200 generations of selective pressure in glucose-, sulfate-, or phosphate-limited chemostats. Chemostats provided a consistent environment across time and replicates, and the nutrient-limited conditions presumably represent a selective pressure encountered by microbes in natural environments, since competition for nutrients is a driving evolutionary pressure that is thought to have shaped the long-term evolution of biological networks [20],[21].

Consistent with earlier work [14], we found that only a few mutations accumulated over the course of our experiments. Most of these mutations appear to be adaptive and provide fitness advantage of 5–10%, although in sulfate-limited evolutions a class of transporter gene amplifications provided a 50% advantage. We found that the outcomes of adaptation can be understood based on the distributions of mutational effects in these different nutrient limitations and the particular population dynamics of our experiments, in which the large diversity of genetic variation generated during the batch phase of growth and clonal interference in the very early stages of our experiments drove the subsequent results.

These factors also determine the level of both genotypic and phenotypic variation between independent populations evolving in response to the same nutrient limitation. We found that the phenotype of adapted individuals, as measured using global gene expression, is much less variable in clones from cultures adapted to sulfate limitation than either glucose or phosphate limitation. This is also reflected in the genotypic diversity among cultures; sulfate adapted clones almost invariably carried amplifications of *SUL1*, which encodes a high affinity sulfate transporter, while the genomes of clones adapted to glucose and phosphate limitation were much more diverse. In some cases, our understanding of the identity and range of these adaptive mutations has led to the realization that expression of particular genes is important to physiology with which the gene had not previously been associated. Thus in addition to answering mechanistic questions about evolutionary mechanism, and the constraints inherent in the system-level organization of metabolic processes in the cell, our work suggests that experimental evolution will also be useful in understanding the function and regulation of individual metabolic pathways.

## Results

We studied 24 prototrophic populations evolving in chemostats in defined media in one of three conditions: glucose limitation, sulfate limitation, or phosphate limitation (Table 1; see Methods). For each limitation, eight populations were founded using ancestors of two strain backgrounds and two ploidies. Population sizes were of order 10^10^ cells. Cultures were maintained for an average of 234±58 generations. At the end of each evolution, two randomly selected clones were isolated from each population for further analysis.

**Table 1:** Summary of experimental evolution experiments.

Population Name	Limitation	Strain Background	Ploidy	Mating Type	Generations of evolution*
G1	glucose	S288c	1N	MATα	182
G2	glucose	S288c	1N	MATa	311
G3	glucose	S288c	2N	MATa/α	238
G4	glucose	S288c	2N	MATa/α	237
G5	glucose	CEN.PK	1N	MATa	164
G6	glucose	CEN.PK	1N	MATα	162
G7	glucose	CEN.PK	2N	MATa/α	237
G8	glucose	CEN.PK	2N	MATa/α	328
P1	phosphate	S288c	1N	MATα	180
P2	phosphate	S288c	1N	MATa	316
P3	phosphate	S288c	2N	MATa/α	222
P4	phosphate	S288c	2N	MATa/α	205
P5	phosphate	CEN.PK	1N	MATa	161
P6	phosphate	CEN.PK	1N	MATα	217
P7	phosphate	CEN.PK	2N	MATa/α	225
P8	phosphate	CEN.PK	2N	MATa/α	201
S1	sulfur	S288c	1N	MATa	297
S2	sulfur	S288c	1N	MATa	188
S3	sulfur	S288c	2N	MATa/α	306
S4	sulfur	S288c	2N	MATa/α	256
S5	sulfur	CEN.PK	1N	MATa	297
S6ˆ	sulfur	CEN.PK	1N	MATα	122
S7	sulfur	CEN.PK	2N	MATa/α	303
S8	sulfur	CEN.PK	2N	MATa/α	250

*: Generation zero is defined as the point at which chemostat flow was initiated.

ˆ A clone from this population, S6c1, has previously been reported in [18]. In that paper the clone was called g122.

We performed 24 independent experimental evolutions in chemostats under three different nutrient limitation regimes. Evolutions were performed using two different strain backgrounds that are amenable to long-term cultivation in chemostats. All strains were wildtype prototrophs.

### The Phenotypic Outcomes of Nutrient-Limited Evolution

In order to characterize the nature and diversity of the adaptive responses to nutrient limitation, we measured culture parameters and gene expression patterns in the final evolved populations and clones derived from these populations.

#### Physiological phenotypes

We detected changes in culture physiology over the course of the evolutionary experiments, consistent with improved fitness. Clones isolated from the evolved cultures and established in independent chemostats displayed similar physiological properties as the populations from which they were derived. 18 of 24 cultures showed an increase by an average of 11% dry weight yield when compared to their respective ancestral cultures. Phosphate- and glucose-limited cultures tended to increase in dry weight more often than sulfate-limited ones, consistent with the smaller proportion of cell mass contributed by sulfate compared to phosphate or glucose [22]. Average cell volume increased in 7 of 8 phosphate-limited cultures, but tended to decrease in the two other nutrient limitations. In the glucose-limited cultures, residual ethanol decreased almost 10-fold in six cultures, as seen in a previous study [17]. However, residual ethanol increased by 60% in two glucose-limited populations, suggesting a different mode of adaptation. Residual glucose decreased in every glucose-limited culture by an average of 45%, consistent with improved extraction of the limiting nutrient. Residual phosphate and sulfate concentrations were below the limit of detection using standard assays even in the founding populations precluding meaningful comparisons.

#### Gene expression phenotypes

To investigate how adaptation was reflected in altered transcriptional programs, we determined global gene expression phenotypes of two evolved clones from each population (N = 48) and a subset of complete evolved populations (N = 15). RNA from evolved and ancestral strains grown in matched conditions in chemostats was co-hybridized to DNA microarrays (see Methods). In order to classify transcripts as significantly altered, we determined the experimental variance due to both growth conditions and microarray methods between two replicate chemostats (see Methods). The majority of genes passed our resultant criterion of >1.5 fold change in two or more expression sets (5443/6237 array features). Those genes that did not change in gene expression were enriched for functions in transcription and signaling (Table S1).

**Figure 1:**
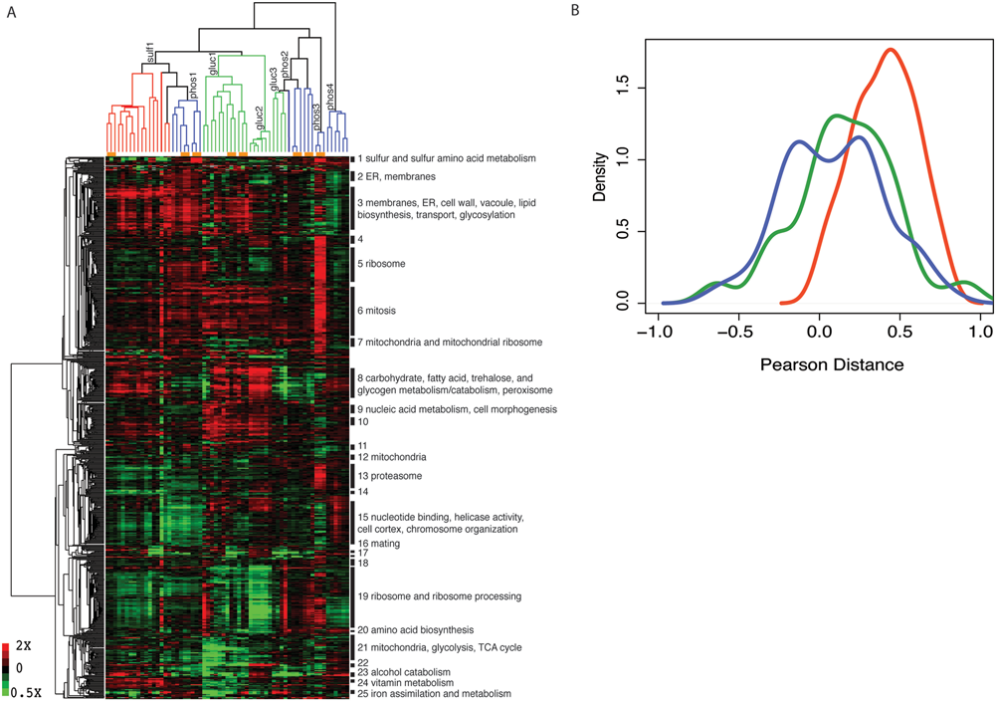
Adaptation to nutrient-limitation results in massive remodeling of global gene expression . (A) Gene expression data, presented as the log_2_-transformed ratio of each gene's expression value in the evolved versus ancestral strain, were hierarchically clustered on both axes (y-axis, 5443 genes; x-axis, 48 clone and 15 population samples). The dendrogram for the clustered experiments (x-axis) is color-coded by nutrient limitation (sulfate-limitation in red, glucose-limitation in green, and phosphate-limitation in blue). Orange horizontal bars represent groupings where the two clones and their corresponding population sample are more correlated with each other than with any other experiments. Glucose expression states fall into three phenoclusters (Gluc1, Gluc2, Gluc3) while phosphate expression states fall into four (Phos1, Phos2, Phos3, Phos4). (B) Density estimates of the distribution of pairwise pearson correlations (N = 112) of the expression states of clones selected under three different nutrient limitations. Clonal isolates from independent sulfate-limitation evolutions (red) were more similar to each other (median pearson distance = 0.425) than those obtained from independent glucose (green, median pearson distance = 0.152) or phosphate (blue, median pearson distance = 0.088) evolutions. The three distributions were compared using the Wilcoxon-Mann-Whitney rank-sum test. The distributions of pairwise correlations between sulfate and glucose clones are significantly different (U = 3097, p-value = 5.9×10^−11^) as are the distributions between sulfate and phosphate clones (U = 2545, p-value = 1.54×10^−14^). The distributions of pairwise distances between phosphate and glucose clones are not significantly different (U = 7103, p-value = 0.08681).

We performed two-dimensional hierarchical clustering of the resulting data matrix (Figure 1A). Unsupervised clustering of experiments resulted in groupings that corresponded to the nutrient limitations, consistent with different selective regimes leading to different gene expression outcomes. The two independent clones from each population typically clustered with each other and with the population sample (orange bars, Figure 1A), indicating that individuals within populations tended to be more similar to each other than individuals from different populations, and that clones are representative of the population as a whole.

To quantify these observations, we calculated the distribution of pairwise pearson correlations for all expression profiles. This metric, which ranges from −1 to 1, provides a measure of the difference between phenotypic states. Positive values indicate similarity between pairs of expression states, while negative values reveal divergent expression states and values near zero indicate the absence of any relationship. Using this measure, we found substantial phenotypic homogeneity in the individual evolved populations. On average the two clones within a population are well correlated in expression (average pairwise correlation greater than 0.70; Table 2). Furthermore, the patterns of gene expression of the clonal isolates were well correlated with the patterns determined for the entire evolved population, supporting their suitability as representative samples of the populations (Table 2).

**Table 2:** Diversity of gene expression phenotypes within and between evolved populations.

Selection	Phosphate	Sulfur	Glucose
Intrapopulation pairwise pearson distance between clones within populations	0.84±0.17 (n = 8)	0.70±0.17 (n = 8)	0.70±0.19 (n = 8)
Intrapopulation pairwise pearson distance between clones and population sample	0.66±0.13 (n = 16)	0.76 (n = 2)	0.68±0.20 (n = 12)
Interpopulation pairwise pearson distance between clones within limitations	0.09±0.31 (n = 112)	0.40±0.20 (n = 112)	0.16±0.32 (n = 112)

We computed pairwise pearson distances. The value presented is the mean of all pairwise pearson distance±one standard deviation. n indicates the number of pairwise distances calculated.

The distribution of the pairwise pearson correlations can also be used to compare the diversity of the patterns of gene expression among the several populations that were evolved independently under the same nutrient limitation. Analysis of these distributions for the three different nutrient limitation regimes (Figure 1B and Table 2) show that there is greatly reduced diversity in the gene expression profiles of clones derived from the independent sulfate-limited populations than is the case for either glucose-limited or phosphate-limited populations. This same result can be seen in the dendrogram at the top of Figure 1A, which shows that the expression states of sulfate adapted populations cluster much more tightly than do either glucose or phosphate adapted populations. The dendrogram also indicates that the several independently evolved glucose and phosphate expression patterns each fall into one of a few apparently distinct phenotypic classes. These data show that adaptation to sulfate limitation appears to be constrained, resulting in a high degree of phenotypic parallelism between evolution experiments, whereas alternative phenotypic outcomes are obtained in response to glucose and phosphate limitation.

#### Metabolic strategies for adaptation to nutrient limitation

One motivation for undertaking experimental evolution studies is to try to understand the adaptive metabolic strategies available to yeast cells. Conceivably, there might be condition-independent efficiencies possible. We could expect to observe these as red or green stripes across all of Figure 1A, representing clusters or genes up- or down-regulated, respectively, in all the evolved cultures. However, since we studied three very different environmental conditions, we might expect most strategies to be condition-specific. Indeed, there are no obvious red or green stripes across the whole of Figure 1A, indicating that there are very few if any clusters of genes that are generally up- or down-regulated in all the evolutions. There are, however, many big blocks of red and green that indicate common strategies for individual selective conditions. Indeed, as suggested above, for both glucose and phosphate limitation these blocks subdivide the evolved cultures into subclasses which differ in the genes whose expression levels have changed over the evolution. It should be noted that Figure 1A provides gene expression patterns uncorrected for differences in gene copy number (see below); thus some of the narrow vertical stripes of changed expression may reflect aneuploidy more than metabolism *per se.*

We performed gene ontology (GO) term enrichment analysis for clusters of genes with correlation coefficients greater than 0.7 that contained more than 25 genes using the program GOLEM [23]. Clusters with significantly enriched GO process terms (hypergeometic test, Bonferroni corrected p-value <10^−5^) are labeled in Figure 1A (closely related GO terms were condensed for clarity, and broad GO terms were omitted, see Table S1 for complete data). It is clear from this analysis that the behavior of genes across adaptations differs among the three different nutrient limitations; that is, there is no evidence of condition-independent adaptive strategies. Table 3 shows a more stringently selected subset of genes showing the greatest increase and decrease in expression in the evolved strains from each nutrient limitation. Some GO categories did increase in more than one condition, such as GO Function category “transmembrane transporter activity,” which is enriched in both glucose and phosphate limitations, and Cellular Component term “integral to membrane,” enriched in phosphate and sulfate. As we document further below, many of the evolved strains overproduce high-affinity transporters that increase the uptake of the limiting nutrient in their environment. Among the genes generally decreased in expression, both glucose and phosphate limitations are enriched for genes in the closely related GO Process categories “tricarboxylic acid cycle” and “generation of precursor metabolites and energy,” which might indicate that cells are functioning more efficiently. Genes annotated to “iron ion binding” and “iron homeostasis” also shared patterns of decreased expression in glucose and sulfate limitations.

**Table 3:** Gene Ontology enrichment analyses of genes showing the greatest increase and decrease in expression following adaptation.

		Glucose		Phosphate		Sulfur	
	Term	p-value	N_class_/N_genome_ (%)	p-value	N_class_/N_genome_ (%)	p-value	N_class_/N_genome_ (%)
**>1.5 SDs**		**n = 230 genes**		**n = 305 genes**		**n = 343 genes**	
**Process**	Cellular lipid catabolic process	0.0056	7/23 (30.4)				
	Sulfur metabolic process			1.39×10^−9^	23/73 (31.5)		
	Response to toxin			6.85×10^−8^	20/65 (30.8)		
	Nitrogen metabolic process			8.25×10^−7^	42/280 (15.0)		
	Oxidation reduction			0.00517	33/268 (12.3)		
	Carbohydrate metabolic process					8.86×10^−9^	47/261 (18.0)
	Vitamin metabolic process					0.00075	19/88 (21.6)
	Cell wall organization/biogenesis					0.00401	30/203 (14.8)
**Function**	Transmembrane transporter activity	0.00051	31/308 (10.1)	0.00037	38/308 (12.3)		
	Oxidoreductase activity			0.00456	35/304 (11.5)		
	Hydrolyzing O-glycosyl compounds					1.25×10^−5^	14/40 (35.0)
**Component**	Peroxisome	0.00825	10/54 (18.5)				
	Integral to membrane			0.00135	90/1065 (8.4)	9.44×10^−6^	107/1065 (10.0)
	Cell wall					8.98×10^−13^	32/103 (31.1)
**<1.5 SDs**		**n = 314 genes**		**n = 292 genes**		**n = 218**	
**Process**	Alcohol biosynthetic process	5.91×10^−10^	20/56 (35.7)				
	Cofactor metabolic process	2.15×10^−8^	36/184 (19.6)				
	Tricarboxylic acid cycle	4.2×10^−8^	14/29 (48.3)	0.0071	9/29 (31.0)		
	Nitrogen compound metabolic process	8.3×10^−7^	43/280 (15.4)				
	Oxidation reduction	2.34×10^−6^	41/268 (15.3)				
	Iron homeostasis	0.0055	10/33 (30.3)			1.47×10^−5^	11/33 (33.3)
	Nucleotide metabolism	0.00093	26/171 (15.2)				
	Vitamin metabolic process	0.00447	17/88 (19.3)				
	Response to toxin	0.0078	14/65 (21.5)				
	Generation of precursor metabolites and energy	0.00381	38/313 (12.1)	0.0048	36/313 (11.5)		
	Amino acid and derivative metabolism					0.00345537	11/33 (33.3)
	Regulation of translation					0.0058	32/358 (8.9)
	Posttranscriptional regulation of expression					0.00915	32/366 (8.7)
**Function**	Oxidoreductase activity	1.18×10^−8^	48/304 (15.8)				
	Iron ion binding	9.79×10^−5^	22/115 (19.1)			5.76×10^−7^	21/115 (18.3)
	Transmembrane transporter activity	0.00015	40/308 (13.0)				
	ATP-dependent helicase activity			3.83×10^−6^	18/71 (25.4)		
	Pyrophosphatase activity			0.00119	37/321 (11.5)		
	Isocitrate dehydrogenase activity			0.00775	4/5 (80)		
	Transition metal ion binding					0.0038	43/519 (8.3)
**Component**	Plasma membrane	0.00012	40/312 (12.8)				
	Phosphopyruvate hydratase complex	0.0071	4/5 (80)				

Genes +/−1.5 SD from the mean of summed expression changes across all experiments were analyzed for statistically significant GO term enrichment by computing a p-value using the hypergeometric distribution (The background set of genes was 5443, the number measured in microarray experiments). Nonredundant, statistically significant enriched GO terms (Bonferonni corrected p<0.01) are listed below. The number of genes annotated to each GO term that were +/−1.5 SD from the mean (N_class_) were divided by the number of genes within the genome that are annotated to that GO term (N_genome_) to determine a fractional representation of each term (%).

Most differentially expressed genes showed increases and decreases in expression in only a single selective regime. Notable in this regard are the strongly reduced expression of genes explicitly involved in fermentation (“alcohol biosynthetic process” in Table 3) in strains evolved in glucose limitation. This recapitulates a similar result obtained by Ferea et al. [17] for yeast strains evolved under glucose limitation. This result, and the concomitant increases in peroxisomal functions (GO Component) and lipid metabolism (GO Process) are consistent with the conclusion of Ferea et al. [17] that adaptation involves major alterations in metabolic strategy. For the strains evolved in phosphate limitation, the notable increases are genes annotated to sulfate metabolism, nitrogen metabolism, oxidation and reduction, and response to toxin. The genes in these subsets are overlapping, and focused on redox-related reactions, for example cytochrome oxidase. The decreases in the Function category include ATP-dependent helicases and pyrophosphatases. For sulfate limitation, the notable increases include overlapping sets of genes involved in carbohydrate and cell wall metabolism, and a highly significant increase in the “cell wall” component category.

In order to assess the coordination in gene expression changes more directly at the level of metabolic pathways, we analyzed gene expression data using the Saccharomyces Genome Database (SGD) Pathways Tools Omic Viewer (http://pathway.yeastgenome.org/expression.html). We found that the three different clusters of glucose evolved clones and populations (Gluc1–3, Figure 1A) corresponded to differing strategies in carbon use. Cluster Gluc1 showed evidence of concerted down regulation of genes involved in both gluconeogenesis and glycolysis. These same genes were unchanged in Cluster Gluc2, which showed increased expression of *CIT2* and *CIT3*, both of which catalyze the synthesis of citrate in the citric acid cycle. Moreover, genes catalyzing fatty acid oxidation (*POX1*, *FOX1*, *POT1*, *DCI1*) were coordinately up-regulated in this cluster. In both clusters Gluc1 and Gluc2, *ADH2*, which catalyzes the conversion of ethanol to acetylaldehyde, was up-regulated. By contrast, in cluster Gluc3 *ADH2* was down-regulated and there was otherwise little change in the gene expression of metabolic enzymes. These gene expression patterns agree with the ethanol measurements described above. That is, the six populations in clusters Gluc1 and Gluc2 showed decreases in ethanol concentration while the two populations in cluster Gluc3 increased ethanol. The divergent responses of central carbon metabolism genes in clones adapted to glucose limitations are indicative of metabolically different evolutionary strategies underlying the adaptive response.

### The Genotypic Responses to Nutrient-Limited Evolution

To identify the range of genotypic responses to adaptation to nutrient-limited environments, we comprehensively characterized the genomes of evolved clones using several microarray-based methods that in combination identify the suite of structural, insertional and nucleotide variants in each clone [20]. This analysis enabled a direct comparison of the diversity of phenotypic outcomes of experimental evolution with alterations in the genome.

#### Specific genomic amplifications including transporter genes

**Figure 2:**
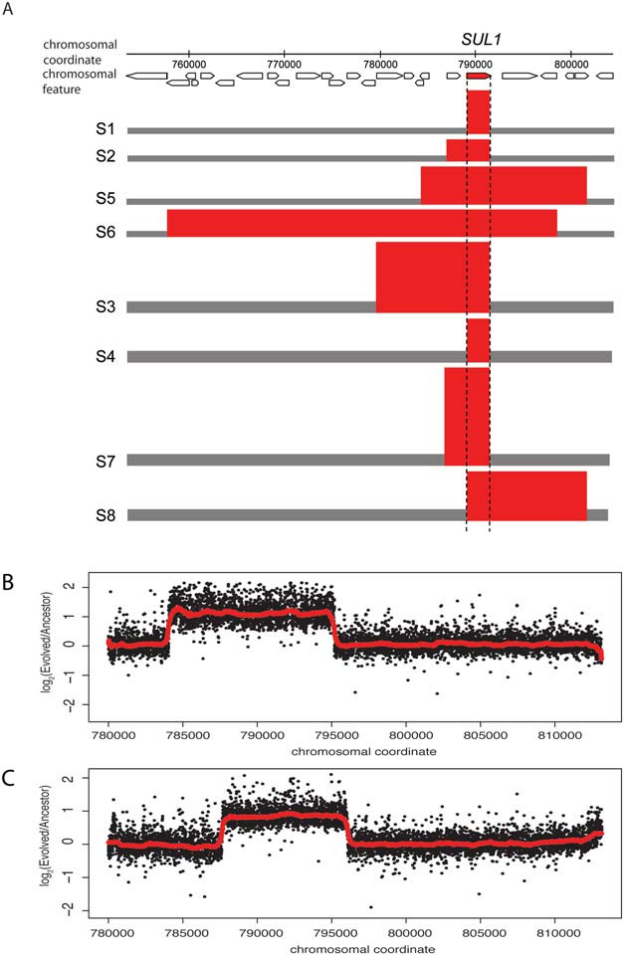
*SUL1* is amplified in multiple independent evolutions. (A) Amplified fragments that include the gene *SUL1* were identified in clones recovered from all sulfate evolutions. Amplicons, in red, span the length of the CGH signal deviation from wildtype ploidy (in gray). The height of the amplicon reflects the copy number relative to wildtype (gray bars, height scaled to haploid or diploid copy number as appropriate). The number of copies of amplified fragments was determined by averaging CGH data from two clones from each population, with the exception of populations S1 and S2, in which only one clone was used due to disagreement between the clones. We analyzed two of these amplicons in further detail using a high density overlapping tiling microarray. The breakpoints for *SUL1* amplifications were precisely mapped in the haploid clone S2c1 (B) and the diploid clone S4c1 (C). Complete data are shown for the ratio between independent hybridizations of evolved and ancestral DNA for the 7356 probes that span chromosomal coordinates 780003-813512 of chromosome II. A running median was computed using the R function *runmed* with a median window width of 201 (red line).

**Figure 3:**
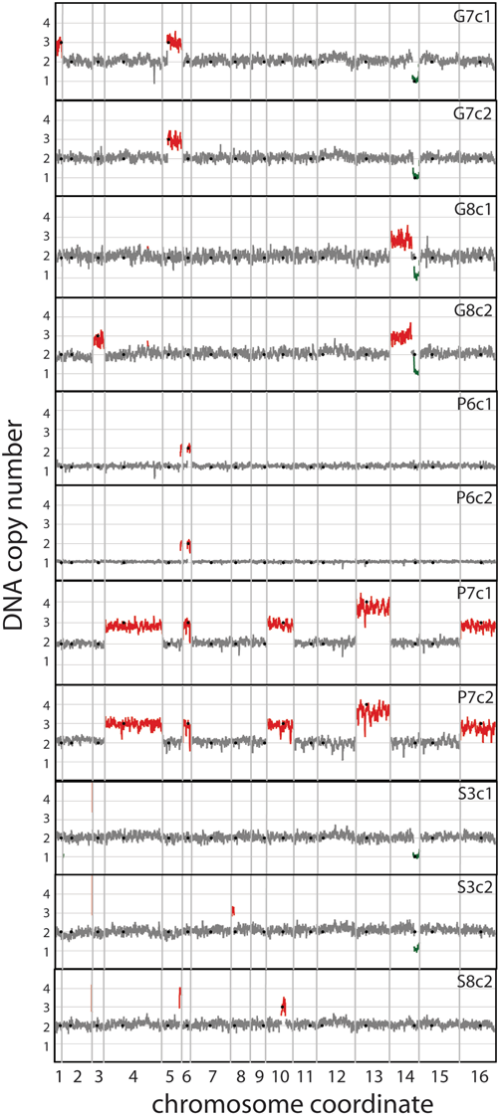
Structural genomic variation is detected in clones selected from all long-term nutrient limitations. We computed a running average of log_2_ ratios between evolved and ancestral genomes determined using CGH across 7 consecutive genes. Contiguous regions deviating from wildtype ploidy levels are colored red for amplifications and green for deletions. Regions that did not deviate from wildtype copy number are in gray. Centromeres of each of the 16 chromosomes are indicated by black dots.

We analyzed structural variation in the genomes of evolved clones using microarray comparative genomic hybridization (CGH). We identified extensive structural variation in the genomes of clones recovered from the three nutrient limitation regimes (Figure 2, Figure 3, Table S2, and Figure S1). One prominent class of structural variation included discrete genome amplification and deletion events. In glucose and sulfate limited selections, amplification of a specific nutrient transporter was a common adaptive outcome. Under glucose limitation, amplifications of either of the adjacent *HXT6* and *HXT7* genes, encoding high affinity glucose transporters, were observed in 9 clones from 5 populations. Under sulfate limitation we observed amplification of *SUL1,* which encodes the high affinity sulfate transporter, in 15 of 16 clones (Figure 2). The single clone without the amplification, S1c1, was later found to contain several point mutations (see below). In phosphate limitations the secreted acid phosphatase gene *PHO5* changed in copy number in both clones isolated from 2 of the 8 populations. Interestingly, in one population this event was an amplification and in the other it was a complete deletion.

Amplification of *HXT6* and *HXT7* has previously been shown to result from unequal mitotic recombination between these neighboring genes, which are 99% identical at the nucleotide sequence level [24]. Our CGH data show amplified signal only at the *HXT6* and *HXT7* probes (which are of identical sequence), consistent with the occurrence of this specific amplification by the same mechanism. By contrast, the amplification of *SUL1* in long-term sulfate selections appears to result from a diversity of events, as CGH data showed a variety of amplified alleles at this locus (Figure 2A). We observed a variety of breakpoints bounding this amplification, but detected no obvious repetitive sequence or genomic features at the amplification boundaries. The number of *SUL1* copies varied from 2 to 16 between individuals, and the amplified fragment ranged in size from just the 2.5 kilobase *SUL1* gene to over 40kb (Figure 2A). To more precisely define the boundaries of the *SUL1* amplifications, we hybridized DNA from two individuals to an overlapping tiling microarray that covers the entire yeast genome with an average of 4 basepair (bp) resolution, allowing us to identify breakpoints to within 100 bp (Figure 2B and 2C).

We searched for identical sequence at these boundaries and found that the longest identical sequence match between the left and right breakpoints was only 7 bp for S2c1 and 6 bp for S4c1. Previously, we had identified minimal sequence homology (3bp) bounding a deletion of the gene *ACE2* that underlies suppression of an *AMN1* knockout [14]. Thus, small stretches of sequence identity at the boundaries of both amplifications and deletions appear to be sufficient to facilitate these events, as recently demonstrated [25].

In all but one case, clones isolated from the same population shared breakpoints. Since this is unlikely to occur by chance, this indicates that mutations found in both clones reflect a single initial event, which then spread to high frequency in the population. *SUL1* amplifications are likely to be present as tandem repeats at the *SUL1* locus, as most strains carrying the amplification showed chromosome II size increases (as detected by pulsed field gel electrophoresis and confirmed by microarray analysis of the shifted bands). The amplification also segregated as a single mendelian locus in two tetrads from evolved diploids and one tetrad from a backcrossed evolved haploid, as determined by CGH (Table S3). Clones sometimes differed in copy number, suggesting that once formed, the tandem repeat can expand further to higher copy numbers within a lineage.

#### Large duplications, deletions, and rearrangements

We detected a second class of prevalent structural variation in evolved clones, consisting of gross chromosomal events. Segmental changes in copy number were detected by CGH (Figure 3) with transposon and tRNA sequences at the breakpoints (Table S2), as previously reported for a different glucose-limited dataset [26]. Entire chromosomes were also found amplified in some strains, including one diploid population (P7, Figure 3), with substantial aneuploidy. Diploids were more likely than haploids to have large amplifications and deletions, in contrast to the transporter gene amplifications, which were observed equally in haploids and diploids. Only two haploid clones, both from the same population, contained amplified chromosome segments, while seven diploid clones from five populations showed such changes. In order to characterize the physical structure of these changes, and to potentially identify reciprocal translocations that would not be detectable by CGH, we ran pulsed field gel electrophoresis (PFGE) on all clones. Novel bands and bands corresponding to predicted translocation sizes were excised from the gel and hybridized to microarrays (Figure S1). In all cases, translocations hypothesized from the CGH data were consistent with the PFGE results (Table S2). Three clones had chromosome XII size polymorphisms consistent with changes in ribosomal DNA copy number (Table S2).

Certain regions of the genome appeared to be particularly susceptible to large-scale genomic events. For example, one copy of the right arm of chromosome XIV was deleted in three diploid sulfate- or glucose-limited cultures (Figure 3) and is associated with a tRNA and LTR-dense breakpoint located between *CIT1* and *ATO2,* as previously reported [26]. In addition, we found two independent uses of a breakpoint between *YNL018C* and *PUB1*, which contains a tRNA. Overlapping segments of chromosome V were amplified in four populations from all three nutrient limitations. Two of these independent amplifications shared identical breakpoints at a Ty1 element. Thus in contrast to transporter gene amplifications, which are invariably specific to one nutrient limitation, repeated gross chromosomal rearrangements were found in multiple nutrient limitation regimes. This suggests that structural variation in these regions may underlie a general adaptive advantage to nutrient limitation or growth in the chemostat. These repeatedly used sites represent structurally flexible loci in the genome that may mediate potentially temporary and reversible genomic alterations [27]. The exact targets of selection in these regions remain unknown, though bulk competition experiments in chemostats have suggested that hemizygosity of a cluster of genes on chromosome XIV, coinciding with our recurrent deletion site, confers a fitness advantage in glucose- and nitrogen-limited chemostats [28].

#### Transposon activity during evolution experiments

In addition to their association with chromosomal rearrangements, transposons are themselves a rich source of genomic variation with important roles in evolution [29],[30]. We used array-based transposon specific extraction (TSE) to identify novel Ty insertion sites using probes targeting Ty1 and Ty2 consensus sequences [15] (see Methods). We analyzed one clonal isolate from each of the 24 evolutions, and identified six new full length retrotransposition events (Table S4). In four cases the exact site of the insertion was confirmed by PCR analysis and/or tiling DNA microarray. Four insertions corresponded to retrotranspositions into genomic sites known to be targets for Ty elements, since they contain tRNAs or LTR sequences [31]. In one case, the new insertion was also the breakpoint of a translocation carried by the clone (G7c1, Table S2). CEN.PK differs in its transposon content from the sequenced S288c strain [15]. However, in two clones evolved from a CEN.PK background new retrotransposition events were identified at a site on chromosome I that contains a Ty in S288c but not in wildtype CEN.PK. This provides compelling evidence of recurrent Ty insertions at particular sites in the genome that can occur over short time scales. We found an additional Ty retrotranposition in a phosphate-limited strain, inserted between a Ty1 delta element and the gene *YPR003C*.

Two retrotransposition events occurred within genes. We identified a novel insertion site on chromosome IX in *MTH1*, a negative regulator of glucose sensing, in the glucose-limited clone G2c2. PCR and sequence analysis of this insertion revealed that it was derived from *YMLWTy1-2*, which is encoded on chromosome XIII. MTH1p is known to regulate the function of the transcription factor RGT1p, which induces expression of the hexose transport genes in the presence of glucose and represses their expression in the absence of glucose [32]. Thus, it is plausible that a downstream effect of the insertion of a Ty in *MTH1* is constitutive activation of *RGT1* leading to increased expression of sugar transporters. Although this model remains to be fully tested, the expression levels of known RGT1p targets, *HXT1*, *HXT2*, *HXT3* and *HXT4,* increase dramatically in this strain (*HXT1*: 5.9 fold, *HXT2*: 3.5 fold, *HXT3*: 1.5 fold, *HXT4*: 6 fold). A second glucose-adapted clone, G6c1, carried a novel Ty insertion in *NUT1*, a component of the RNA polymerase II mediator complex.

#### Single-nucleotide mutations acquired during evolutions

To identify single-nucleotide changes (SNPs) accumulated over the course of the evolutions, we hybridized DNA from 10 evolved clones to overlapping tiling microarrays and used the SNPScanner algorithm to detect candidate mutations [14]. These samples included haploid and diploid clones from nine populations of the S288c strain background adapted to all three nutrient limitations. To identify adaptive pathways that did not require amplification of transporters or other gross chromosomal rearrangements, we preferentially selected clones for analysis that yielded negative CGH results. Thus, of the 10 clones analyzed for SNP variation only two clones had known structural variation, both of which were recovered from sulfate evolutions (S2c1 and S4c1).

We sequenced predicted SNPs using targeted Sanger sequencing reactions. In total we confirmed 34 mutation events in ten clonal isolates (Table 4). Based on previous assessment of the SNPScanner algorithm we expect to detect ∼85% of SNPs genome-wide [14]. Thus, it is likely that an additional six mutations remain undetected among these 10 genomes (i.e. a false negative rate of less than one SNP per genome on average). One SNP prediction corresponded to the aforementioned Ty insertion in *MTH1* in clone G2c2. An additional prediction was found to result from a 338bp LTR insertion within *SNF6* in clone G1c2. On the basis of sequence we surmised that this event is likely the result of retrotransposition of a full length Ty1 and subsequent recombination event resulting in a solo LTR (Figure S2). Of the 32 single base pair mutations, 27 (84%) occurred in known or predicted genes and 5 (16%) were in intergenic regions. Although the total number of mutations in each clone is small, most clones had a similar number of point mutations. A notable exception was clone G1c1, which had three times as many point mutations as the mean of all clones. A possible explanation for an increased mutation frequency in this clone is provided by the identification of the F77S mutation in *MMS2*, a component of the error-free post-replication repair pathway. A null mutation in *MMS2* has been estimated to result in a tenfold higher spontaneous mutation frequency over wildtype [33]. However, we were unable to detect an increase in mutation rate in clone G1c1 using fluctuation analysis (data not shown).

**Table 4:** Genome-wide nucleotide variation in evolved clonal isolates.

Population	Clone ID	SNPs	Intergenic	Genic	Synonymous	Missense	Nonsense	Alleles (% in final population±95% CI)
G1	G1c1	9	1	8	2	6	0	*CCR4* E724V (29±3)
								*PTH2* I138M (26.5±0.3)
								*GIN4* L1079M (22±6)
								*BRR2* G524S (28±1)
								*MMS2* F77S (23±4)
								*SAP185* synonymous (39±5)
								*CST9* synonymous
								*YNR071C* S177L
								Intergenic between *SPP2* and *SMP3*
G1	G1c2	1	1	0	0	0	0	*SNF6* LTR insertion (1.7)
								Intergenic between *AGC1* and *YPR022C*
G2	G2c2	4	0	4	0	3	1	*TFC3* G877K
								*SIM1* A317V
								*WHI2* Q228X
								*YMR185W* A926V
								*MTH1* retrotransposon insertion
G4	G4c1	3	0	3	0	2	1	*GSH1* D188F (100)
								*BNI5* R367I
								*RIM15* S1580X
P1	P1c2	2	0	2	0	2	0	*SIR1* C135Y (56±8)
								*CKA2* D186N (60±11)
P2	P2c2	1	0	1	1	0	0	*KGD1* synonymous
P3	P3c2	3	1	2	0	2	0	*SLH1* E193K (100±8)
								*URB2* E1018D
								Intergenic between *YGL258W*-A and *YGL258W*
S1	S1c1	3	0	3	2	0	1	*SGF73* E277X (22±1)
								*ERG1* synonymous (20±2)
								*SFP1* synonymous
S2S2	S2c1S2c1	3	0	3	0	1	2	*SGF73* E294X (15±2)
								*UPF3* G6W
								*PBP2* Y127X
S4S4	S4c1	3	2	1	0	1	0	*YEL007W* Y81D
								Intergenic between *AGP1* and *YCL023C*
								Intergenic between *YCR006C* and *tP(AGG)C*
**Total**		**32**	**5**	**27**	**5**	**17**	**5**	

We predicted the presence of SNPs using the SNPScanner algorithm on tiling microarray data. In order to confirm the prediction and identify the sequence change we sequenced the locus using PCR and Sanger sequencing.

Of the 27 mutations that occur within genes, 22 (81%) result in nonsynonymous codon changes or truncating mutations. Notably, no mutations were found in genes encoding transporters or with obvious connection to nutrient import in the cell. No significant gene ontology (GO) term enrichment was found for the 23 different genes in which nonsynonymous SNPs or insertion events occurred. A number of mutated loci in glucose evolved clones have known roles in carbon metabolism: *CCR4, MTH1* and *SNF6* are involved in transcriptional regulation in response to carbon sources [34],[35], and *RIM15* is a protein kinase that mediates entry into the G_0_ stage of the cell cycle in response to nutrient availability [36]. However, in the majority of cases a connection to enhanced growth under nutrient limitation was not apparent on the basis of prior knowledge, thereby requiring additional avenues of investigation (see below). One gene, *SGF73*, which encodes a component of the SAGA complex and has been shown to regulate histone ubiquitination status [37], was mutated in two independent sulfate evolutions in which we recovered two different nonsense alleles. This provides compelling evidence that loss of function or truncation of SGF73p is advantageous under sulfate limitation. Moreover, the detection of independent mutations in the same gene from different sulfate limitation evolutions is consistent with the reduced flexibility in phenotypic and structural genomic outcomes of sulfate selection as compared with glucose and phosphate selection.

### Measurements of Mutant Frequency, Dynamics and Fitness

We investigated the extent to which selection drove the observed genotypic changes and the dynamics by which adaptation occurred.

#### Analysis of allele frequencies through time

In order to measure the frequency of genomic rearrangements and to possibly detect structural variants not identified in the selected clones, we subjected DNA extracted from eleven population cultures to CGH. We detected measurable changes in segmental copy number in five population samples (Figure S3). In two cases (P6 and P7) the changes found in the population sample matched the CGH profile of the corresponding clone samples. Three population samples (G6, P3 and P5) contained high frequency rearrangements not identified in the clones, suggesting that these populations contain additional genetic diversity not identified in our analysis of clonal isolates. We inferred the frequencies of these rearrangements by computing the ratios of the heights (or depths) of the peaks in the population and clone CGH results. In this way we found frequencies of rearrangements within these populations ranged from 17–77%. Interestingly, the copy number change that reached the highest frequency in any population was the massive aneuploidy found in population P7. Because of the difficulty in defining the allele frequency of the variable copy number transporter amplifications, they were excluded from this analysis.

In order to assess the frequencies of point mutations, we developed a quantitative sequencing protocol (Figure S4 and Methods). We validated the accuracy of this approach by independently analyzing clonal isolates from population samples using targeted Taqman allelic discrimination assays for a subset of SNPs (Figure S5 and Methods) and found that the methods produced comparable data (Figure S6). We detected 13 of the 32 SNPs at measurable frequencies (i.e. an allele frequency greater than 5% in the final population, hereafter referred to as significant mutations; Table 4) and focused our investigation on these mutations, as they are more likely to be adaptive. In addition, we tested the frequency of the *SNF6* and *MTH1* insertions by PCR analysis of 96 clones from each population and found that they were present at low frequencies in the population (1% and <1% respectively). We detected two point mutations fixed in populations (a mutation in *GSH1* in clone G4c1 and a mutation in *SLH1* in clone P3c2). Otherwise, significant mutations were found at frequencies well below fixation (15–60%). Notably, when multiple significant mutations were found within a clone (e.g. G1c1 and P1c2), they were found at similar frequencies.

**Figure 4:**
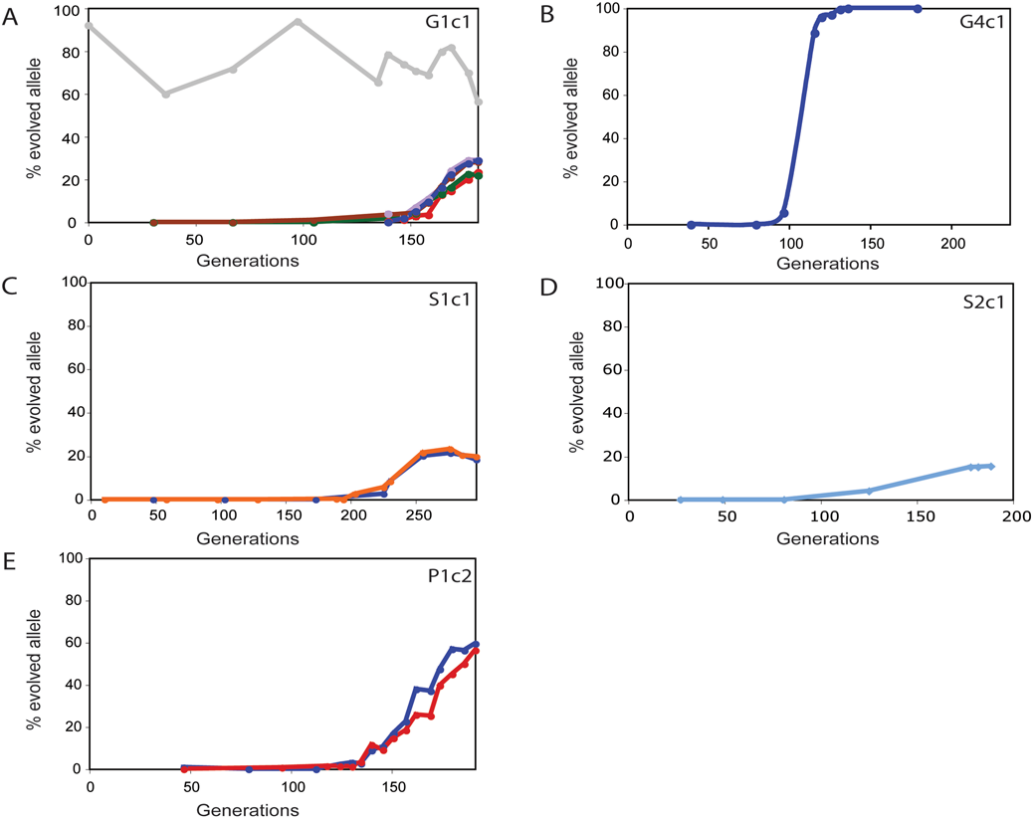
Dynamics of allele frequencies in evolving populations. We determined allele frequencies for SNPs identified at detectable frequencies in the final population sample using quantitative sequencing. (A) clone G1c1 (red, *MMS2*; purple, *SAP185*; green, *GIN4*; brown, *BRR2*; blue, *CCR4*; gray, *PTH2*), (B) clone G4c1 (blue, *GSH1*), (C) clone S1c1 (blue, *ERG1*; orange, *SGF73*), (D) clone S2c1 (blue, *SGF73*) and (E) clone P1c2 (blue, *CKA2*; red, *SIR1*). We computed fitness coefficients for clones using allele frequency estimates as estimates of clone frequencies. Fitness coefficients were calculated for each allele (Table 5) and for each clone as described in methods. Relative selection coefficients of clones are (mean±95%CI): clone G1c1 = 1.0918±0.0159, clone S1c1 = 1.0361±0.0139, clone S2c1 = 1.0247±0.0975, clone P1c2 = 1.0602±0.0081. A selection coefficient for the diploid clone G4c1 could not be computed due to the atypical allelic profile of the *GSH1* mutation.

To ascertain the history of each mutation through the experiment, we determined allele frequencies in archived population samples throughout the course of the evolution for the six populations in which we identified significant mutations (Figure 4). We found that these mutations typically did not reach detectable frequency (i.e. between 1–5%) until more than 100 generations had passed. Their frequency increased thereafter. On the basis of the rate of increase in allele frequencies over time, we computed the fitness coefficient of each mutation relative to wildtype fitness, which is equal to 1. We found significant relative fitness coefficients for the combinations of alleles ranging from 1.0477–1.0918 (Table 5).

**Table 5:** Selective advantage of alleles during evolutions.

Clone	Mutation	Selection Coefficients (coefficient±95% CI)	Generations to maximum frequency	Average final allele frequency	Estimated proportion at t = 0	Subpopulation size at t = 0
**G1c1**	*CCR4* E724V	1.126±0.058				
	*GIN4* L1079M	1.076±0.029				
	*BRR2* G524S	1.074±0.021				
	*MMS2* F77S	1.101±0.023				
	*SAP185* synon	1.067±0.020				
	**Aggregate**	**1.0918±0.0159**	181.5	26.2	2.06×10^−8^	2000
**S1c1**	*SGF73* E277X	1.045±0.013				
	*ERG1* synon	1.063±0.360*				
	**Aggregate**	**1.0477±0.0244**	276.7	20.9	4.90×10^−7^	50000
**S2c1**	*SGF73* E294X	1.025±0.098	188.5	15.55	1.75×10^−3^	1.75×10^8^
**P1c2**	*SIR1* C135Y	1.069±0.0107				
	*CKA2* D186N	1.068±0.0130				
	**Aggregate**	**1.0683±0.0081**	191.5	57.9	2.87×10^−6^	2.87×10^5^

*: not statistically significant.

We determined allele frequencies during the populations’ histories. In order to determine the maximum fitness advantage attributable to the identified mutations we identified the generation at which the allele frequency was the greatest and determined fitness coefficients based on the rate of allele frequency increase to that point. Assuming the fitness benefit has been constant over the evolution experiment we inferred the size of the subpopulation at the commencement of the evolution experiment.

While most allele frequencies increased monotonically, we observed three anomalous alleles. A mutation in *GSH1* was found homozygous at 100% frequency in the final population of a diploid evolution. This mutation rapidly swept to fixation in the population (Figure 4B). Its presence at fixation in this diploid population indicates that it underwent a homozygosing event (e.g. gene conversion). We also identified two cases in which a mutation is present at high frequency in the final population due to a jackpot event in the initial batch phase of growth: a mutation in *PTH2* in clone G1c1 (Figure 4A), and a mutation in *SLH1* in P3c2, which was present at allele frequency of 100% in samples taken throughout the population's history (data not shown). These mutations were either strongly selected during batch phase growth or were neutral but occurred early in the batch phase and subsequently varied in frequency due to genetic drift and/or noise in the quantitative sequencing method. These cases highlight the necessity of retrospective determination of allele frequency dynamics to interpret the final adaptive outcome.

#### Assessment of increased fitness by direct competition

We sought to directly verify the selective advantage conferred by mutations in evolved clones. To do so, we performed fitness assays for two informative cases by competing mutant strains against the ancestral strain (see Methods). We computed fitness coefficients from the rate at which the mutant strains increased in frequency in these competition experiments (Figure S7). We determined that a clonal isolate containing only single nucleotide mutations, G1c1, had a relative selection coefficient of 1.106±0.012 when competed against the ancestral strain. This is consistent with the relative selective advantage for the mutations present in this clone estimated from their rate of increase over the course of the evolution experiment (i.e 1.092±0.016, see Table 5). To confirm the selective advantage of copy number amplification of *SUL1* under sulfate limiting conditions, we engineered a wildtype strain carrying multiple copies of *SUL1* and competed it against the ancestral strain in a sulfate-limited chemostat. Based on these data we computed a relative selection advantage of 1.49±0.09 conferred by multiple copies of *SUL1*.

For strains containing multiple significant point mutations, we sought to determine which mutations are adaptive, which are neutral hitchhikers, and which may interact epistatically. Competition among segregants derived from a backcross breaks the whole genome linkage imposed by asexual propagation and allows for comparison of all combinations of alleles. This results in a mixed population: if there are only two unlinked mutations of interest, the probability of having just one mutation in a segregant is 0.5 and the probability of having both or neither is 0.25. We backcrossed P1c2, in which we had identified mutations in *SIR1* and *CKA2*. Segregants were pooled according to mating type (to avoid the possibility of mating during growth), inoculated into two different phosphate-limited chemostats, and sampled every 2.5 generations. We determined allele frequencies using quantitative sequencing. In both competition experiments, strains carrying the *CKA2* mutation quickly fixed, while those with *SIR1* mutations did not change in frequency, indicating the evolved allele of *SIR1* is neutral and the evolved *CKA2* allele is adaptive (Figure S8). This result would also seem to exclude the possibility that an epistatic interaction between *CKA2* and *SIR1* confers a greater fitness advantage than *CKA2* alone. CKA2p is a catalytic subunit of casein kinase II implicated in a diverse range of biological processes including cell growth, transcription, and flocculation (reviewed in [38]), but with unknown significance to improved growth under phosphate limitation.

### Evolutionary Dynamics of Adaptation

#### Estimating when adaptive mutations appeared in populations

Given the inferred selection coefficients and the observed frequencies of mutations over the course of the evolutions, we can estimate the time at which adaptive mutations must have occurred. Specifically, the frequency of a mutation at time t, p(t), increases as 
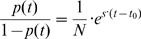
 where s is the increase in fitness conferred by the mutation and t_0_ is the time at which it arose [39]. Population sizes in our experiment are about 10^10^ individuals, and we found that mutations with selective advantage on the order of 10% are initially detected after 100 generations of chemostat evolution, when they reach a frequency of 1% or greater. This implies that at time 0, defined as the point at which chemostat flow is initiated, these alleles were already present at appreciable frequencies. Thus, over the timescales of our experiments, selection primarily acts on standing genetic variation that already existed when selection under continuous culture conditions was initiated. This result is consistent with the yeast evolution experiments of Paquin and Adams [40], who found selection advantages on the order of 10% for mutant clones appearing at detectable frequencies as early as 50 generations; reanalysis of these experiments by Dykhuisen [41] and Otto [42] emphasized that these numbers were not consistent with a simple selection model on mutations that occurred after the start of chemostat flow.

Based on our measurements of the selective advantages of mutant clones, which except in sulfate limitation ranged from 5–12%, and the times at which they reached detectable frequencies, we estimate that the mutations must have been present in 10^2^ to 10^6^ cells (depending on the specific mutation) at the initiation of chemostat flow (Table 5). This means that the mutations occurred 13 to 26 generations after the foundation of the lines in the batch phase of growth, with the earlier-occurring mutations corresponding to those of smaller fitness effect. This implies total mutation rates ranging from 10^−5^ for the early-occurring smaller-effect mutations to 10^−8^ for the later-occurring large-effect mutations.

In these calculations we have assumed that the fitness advantage of a mutant allele is constant over the course of the evolution. Yet we have calculated this fitness advantage on the basis of allele frequency data from when the mutant is relatively common. We cannot rule out the possibility that the mutations have frequency-dependent effects, such that the fitness advantage of a mutant is greater when it is rare than when it is common. It is also possible that the effective fitness advantage of a mutant declines as that mutant becomes more common because other beneficial mutants in competing backgrounds are also becoming more common. If this were the case, then mutations may have occurred later in the experiments than we have estimated. More sensitive allele frequency measurements will help resolve these questions.

## Discussion

Despite the importance of evolutionary ideas in every aspect of biology, there has been relatively little direct experimental data describing the processes and mechanism that underlie evolution. Only recently, through rapidly advancing genome technology, has it become practical to study directly the genetic basis of evolutionary change in an experimental setting. In this study, we analyzed experimental evolution in chemostats with DNA microarray technology, to assess genome-wide variation in gene expression and DNA copy number, and with a practical and affordable method for detecting single-nucleotide changes relative to the sequences of our starting yeast strains. We used these tools to begin to understand the phenotypic and genetic changes characteristic of the evolution of yeast in response to consistent glucose, sulfate, and phosphate limitation in the chemostat.

The main finding of our study is the nature, identity and dynamics of the mutations that occur over the course of these evolution experiments. These mutations confer a selective advantage ranging from ∼5% to as much as 50% per generation. One prevalent class of mutations consists of massive structural genomic alterations, consistent with our earlier observations [26]. The adaptive advantage of a subset of these is readily explained by the fact that amplified genomic segments contain genes encoding transporters that are specific to the applied nutrient limitation. The prevalence of these mutations in adapted populations, the repeatability of their occurrence in independent populations, and the experimental demonstration of their fitness advantage argue for a central role of gene amplification in adaptation to nutrient limitation.

The majority of structural variation, however, is found in other regions of the genome. The reasons for the selective advantage of these variants are less clear, but their repeated observation points to their adaptive value. Interestingly, a recently reported mutation accumulation experiment in yeast also revealed significant aneuploidy in mutation accumulation experiments [43] despite the fact that anueploidies typically cause growth defects [44]. In our experiments a large fraction of these events are clearly associated with the repetitive sequence found in retrotransposons, which are themselves active during these evolutions. However, retrotransposon sequences are not necessary for the generation of structural variation, as illustrated by the repeated but diverse amplifications of the *SUL1* locus. These results point to a structural plasticity of the yeast genome, operating at the supragenic and genic [45] level, that facilitates adaptation.

Whole genome resequencing using tiling microarrays and sanger sequencing revealed that only a small number of point mutations accumulate during these experiments. We estimate that we have found >85% of these mutations, and as new technologies develop, we hope to eventually detect all mutations, including those in difficult repetitive sequences. Our number of acquired mutations is consistent with other microbial experimental evolution studies that have attempted to comprehensively identify mutations. Using a combination of microarray- and mass spectrometry-based sequencing, Herring et al. [5] found a total of 13 mutations in five *E. coli* populations propagated for ∼660 generations using serial transfer. Pyrosequencing a “cooperator” strain of *Myxococcus xanthus* which appeared after 1000 generations of selection identified 15 point mutations [4]. Thus, it would appear that adaptation of microbes proceeds without the requirement for mutator phenotypes in these experiments.

The relative merits of haploid and diploid states with respect to adaptation have been hotly contested [6],[40],[46],[47]. We find an enrichment for gross chromosomal rearrangements in diploid cells as compared with haploid cells, possibly reflecting more deleterious effects of chromosomal rearrangements in haploids [48]. Although we did not explicitly test the rate of adaptation in haploid and diploid cells, our results highlight an underappreciated mechanism by which recessive alleles can be important for adaptation of diploid organisms: namely, through homozygosing via gene conversion or chromosomal aneuploidy. We did not observe any striking differences in adaptation related to mating type or strain background, although it is noteworthy that in all clones from the CEN.PK strain background we identified a *HXT6/7* amplification whereas this was found in only one of eight glucose-limitation adapted clones in the S288c background. Recent analyses have indicated that CEN.PK is a mosaic of S288c genome background and divergent sequence [49]. The genomic region containing *HXT6/7* is identical in S288C and CEN.PK indicating that this observed difference in rates is not due to sequence.

We inferred that the batch phase of growth has a large effect on the parallelism of evolutionary paths in our experiments. During the initial batch phase of growth, the population size doubles every generation, which tightly constrains the time at which mutations occur and means that beneficial mutations are very unlikely to be lost by genetic drift. For example, if there is a class of mutations with a total mutation rate such that on average one such mutation will typically occur after 13 generations, such a mutation will almost always occur sometime between generation 10 and 16. However, when a mutation occurs later than average, it will be present at much lower frequency at the end of batch phase, and hence take substantially longer to spread through the population. The length of this delay depends dramatically on the fitness effect of the mutation: a mutation providing a 50% fitness advantage which occurs 3 generations later than average in batch phase will take 3 extra generations to reach a population frequency of 5%; a mutation of 10% advantage will take 20 extra generations, and a mutation of 1% advantage will take an extra 200 generations.

It follows that if a beneficial mutation that provides a large fitness advantage (of order 50%) occurs at a high enough rate to happen during the batch phase, it will almost always reach a substantial frequency within the population by the end of our experiment. On the other hand, a beneficial mutation of small fitness effect (of order 1%) will typically not do so unless it happens to occur very early in the batch phase (and even in this case if a larger-effect mutation occurs much later, the larger-effect mutation can reach high frequency more quickly). A mutation with effect of order 10% is an intermediate case; it will only reach substantial frequency within the population by the end of our experiment if it occurs early enough in batch phase. However, if mutations of roughly this effect occur at a rate of 10^−7^ or more, at least one such mutation will almost always occur early enough in batch phase to be observed at substantial frequency by the end of our experiment. In this case, the mutation that happens to occur first will typically be the one we observe. In other words, if beneficial mutations of large effect (of order 50%) are sufficiently common that they occur during batch phase, they will almost always occur and take over regardless of what smaller-effect mutations are already present. On the other hand, if large-effect mutations are rare, then the first beneficial mutation of intermediate effect (of order 10%) will typically dominate, because later mutations (even of slightly larger fitness effect) will be at large initial numerical disadvantage.

These dynamics appear to explain the extent of parallelism we observe between populations. In the sulfate-limited evolution, there is a class of *SUL1* amplifications that provide a very large selective advantage (of order 50%). Given this large selective advantage, it is unsurprising that these mutations are observed in almost all of our cultures. This does not necessarily imply that there is only one adaptive pathway in sulfate-limited conditions; it could be the case that there are multiple other mutations which provide alternative ways to adapt to sulfate limitation, but that each of these only provides a selective advantage of order 10% and hence are always eliminated by clonal interference, or are present at much lower frequencies (as is the case for clone S1c1, the only sulfate-limitation adapted clone without a *SUL1* amplification in our study). On the other hand, the phosphate and glucose-limited populations exhibited a broader range of evolutionary responses. In these conditions, we infer that there is no large-effect mutation that is readily accessible, so the result is more dependent on which of the various mutations that confer fitness advantages of order 10% happens to occur first. The fact that we commonly observe certain genomic amplifications (e.g. *HXT6/7*) in these populations suggests that they occur with a total rate comparable to that to the adaptive single point mutations, and confer a similar selective advantage. This argument is consistent with an earlier report that the relative selective advantage of a *HXT6/7* amplification in glucose-limited chemostats is 1.094 (Brown et al., 1998).

Our observations point to an important principle for adaptive phenomena in natural populations and disease: the diversity of adaptive outcomes will vary as a function of the distribution of fitness effects of beneficial mutations, which differs dramatically depending on the selective pressure. If there is a single “solution” that confers a vastly greater selective advantage, that path will be repeatedly observed. Conversely, a diversity of equally beneficial “solutions” will result in a reduction in the reproducibility of adaptation. An illustrative example of this principle is the recent report of selection for resistance of lung cancer cells to gefitinib or erlotinib using longterm culturing of cells in the presence of the drugs [50]. Resistance in one-quarter of the specimens could be attributed to amplification of the oncogene *MET,* implying that alternative routes to resistance must exist that confer comparable fitness advantages to these tumor cells. It is interesting to consider an approach in both our microbial system and cancer studies of blocking known routes to adaptation in order to enrich for unknown alternative adaptive paths that may confer smaller fitness advantages.

Our experiments have identified the outcomes of adaptation to defined environments and revealed the diversity of genomic variation in clonal representatives of adapted populations. Our findings suggest a number of questions that should be addressed. First, it is critical to determine a neutral mutation rate for genome amplifications, deletions and rearrangements as well as the neutral mutation rate for retrotransposition. A recent report has provided new insights into these rates, indicating that large genome events occur at much greater frequencies than nucleotide changes [43]. Cells grown in a chemostat grow at a much slower rate than cells grown at maximal rates in batch cultures, the condition under which mutation rates are typically determined. Evidence in bacteria suggests that single base pair mutation rates are increased under slow growth conditions [51] and in stationary phase [52]. Therefore, examining the rates at which all classes of genomic variants are generated in chemostat cultures will be informative for interpreting future experiments. Second, extending the duration of selection experiments will shed light on the role of subsequent diversity generated during the continuous phase of growth. It will be of great interest to test whether population diversity increases or becomes increasingly constrained as selection continues. Third, varying population size can be expected to have profound effects on the dynamics of adaptation, and can influence the degree of parallelism between independent cultures [9],[10],[53]. This parameter and others such as growth rate and the complexity of the selective pressures will be fertile areas of investigation. Finally, the mechanisms by which these mutations increase fitness and change gene expression will give insight into the functions of these genes and the cellular systems in which they act.

## Materials and Methods

Detailed protocols can be found at http://dunham.gs.washington.edu/protocols.shtml.

### Chemostat Culture

Continuous cultures were established using published methods [54] with the exception of the phosphate-limited media, which contained the following (per liter): 100 mg calcium chloride, 100 mg sodium chloride, 500 mg magnesium sulfate, 5 g ammonium sulfate, 1 g potassium chloride, 500 µg boric acid, 40 µg copper sulfate, 100 µg potassium iodide, 200 µg ferric chloride, 400 µg manganese sulfate, 200 µg sodium molybdate, 400 µg zinc sulfate, 1 µg biotin, 200 µg calcium pantothenate, 1 µg folic acid, 1 mg inositol, 200 µg niacin, 100 µg p-aminobenzoic acid, 200 µg pyridoxine, 100 µg riboflavin, 200 µg thiamine, 10 mg potassium phosphate, and 5 g glucose.

Experiments were started by initially growing cultures in 300mL of the appropriate defined media in batch phase. Once the cultures reached saturation, chemostat flow was initiated. Cultures were grown at a dilution rate of 0.17 volumes/hour. Daily samples were taken from the overflow in order to determine optical density at 600 nm, cell count and viability; perform microscopy; and make archival glycerol stocks. We confirmed that all evolved haploid clones maintained the same mating type as the founder by backcrossing the evolved strain to the isogenic ancestral strain of the opposite mating type. Clones from three of the twelve evolved diploid populations exhibited reduced sporulation efficiency, but did not mate inappropriately.

### Microarray Data

#### Gene expression analysis

Chemostat samples were harvested by fast filtration and frozen immediately in liquid nitrogen. Gene expression differences can be caused by differences in strain background, mating type, ploidy, and nutrient limitation in cultures [55]–[57]. Thus, in order to identify only changes that accumulated over the course of the evolution, we performed all microarray hybridizations of evolved strains using the appropriate ancestral strain grown under matched conditions. RNA was prepared by acid phenol extraction, labeled using a modification of the Agilent low RNA amplification kit, and hybridized to Agilent 60-mer yeast ORF expression microarrays as described previously [44]. Data were acquired using an Agilent scanner and feature-extracted with the Agilent software using default settings. Resulting ratios (transformed to log_2_ values) were filtered for significant signal in at least one channel and renormalized using only genes present at euploid copy number. All raw microarray data are available from the Princeton Microarray Database (http://puma.princeton.edu) and GEO (http://www.ncbi.nlm.nih.gov/geo/query/acc.cgi?accGSE13435). Processed data for expression and CGH microarrays are available as supplementary material (Table S5 and Table S6 respectively).

To obtain a measure of experimental variation in cultivation and microarray procedures we established two independent cultures under identical conditions. We co-hybridized labeled mRNA from both cultures to an expression microarray and analyzed the distribution of the ratios at each microarray feature. The mean of the normally distributed data was equal to zero with a standard deviation of 0.19 log_2_ units. We defined a threshold of three standard deviations, corresponding to a 1.5-fold expression change (i.e. ±0.585 log_2_ units), for significant gene expression changes, consistent with previous reports for chemostat experiments [44].

#### Microarray analysis: CGH

CGH in Figures 3, S1 and S3 was performed as described [44] using Agilent microarrays containing one 60-mer per ORF. CGH in Table S3 used Agilent 44k microarrays containing 60-mers spaced at ∼280 base spacing across the yeast genome.

#### Microarray analysis: PFGE CGH

PFGE and gel band DNA extraction was run as described [26] using double-lane plugs. Resulting DNA was labeled directly using the Invitrogen Bioprime kit and hybridized vs labeled wt genomic DNA as described for CGH.

#### Microarray analysis: TSE

Transposon Specific Extraction (TSE) was performed as described [15] using Agilent 44k microarrays. Extracted samples were hybridized vs matched extracted ancestor DNA, or against total ancestral genomic DNA as indicated in the experiment annotations.

#### Microarray analysis: tiling arrays and SNP identification

Affymetrix Yeast Tiling Array 1.0R arrays were hybridized with total genomic DNA and analyzed as previously described [14]. In order to detect SNPs in diploid samples we analyzed the four spores from a single tetrad. We identified mutations in all four resulting haploids and pursued only those that showed 4:0 or 2:2 segregation in the tetrad. Heterozygous mutations were confirmed in the diploid clones. Because mutations can accumulate during passaging of the cells after the evolution experiment has ended we required that mutations identified in purified DNA were sequence-confirmed in a PCR product obtained by directly amplifying DNA from the glycerol stock using standard colony PCR protocols.

The raw data files (.CEL) are available at http://genomics-pubs.princeton.edu/ExperimentalEvolution/Download.shtml. The SNPScanner result files can be downloaded at this same site and are formatted as .sgr files for viewing in the Integrated Genome Browser (http://www.affymetrix.com/support/developer/tools/download_igb.affx).

### Fitness Competitions

Clonal competition assays were performed using two different drug resistant markers. For testing the fitness of the haploid clone G1c1 a spontaneous canavanine drug-resistant mutant (Can^R^) was selected. Two 300 mL chemostats were inoculated with either the evolved strain marked with Can^R^ or the ancestral strain, which is sensitive to canavanine (Can^S^). Cultures were brought to steady-state conditions over a period of ∼10 generations. 15 mL from the chemostat containing the ancestral strain was removed and replaced with 15 mL from the chemostat containing the Can^R^ marked clone, corresponding to an initial population mix of 5% evolved clone and 95% ancestral clone. We sampled the chemostat an average of every 3 generations for approximately 50 generations. Cells were sonicated, diluted and plated on rich nonselective media and grown for 2 days at 30°C. We counted >200 colony forming units using sterile methods. Cells were then replica-plated to synthetic complete minus arginine media containing 60mg/L canavanine and allowed to grow at 30°C for 3 days. Can^R^ cells were identified as fully formed colonies.

To test the fitness increase due to amplified copies of *SUL1* we performed the same assay. However, in this case we transformed a ura3 strain isogenic to the ancestral strain with a multicopy *URA3* plasmid containing the *SUL1* gene [58]. We constructed a G418-resistant (kanMX) version of the ancestral strain by knocking out the dubious ORF *YDR032W* with the kanMX cassette amplified from the systematic deletion collection. For this competition cells were first plated on YPD and then replica-plated onto YPD+200mg/L of G418.

As a control, we marked the ancestral wildtype strain with Can^R^ and competed it against the isogenic Can^S^ ancestor. This analysis revealed a slight relative fitness advantage of Can^R^ in glucose limiting chemostats of 1.015. Given that this selective advantage is an order of magnitude less than that determined for the evolved clone we considered this contribution to fitness to be negligible.

We calculated the proportion of evolved cells (p) in the population and determined the proportion of ancestral cells q = 1−p. Fitness coefficients were computed by regressing ln(p/q) against the number of generations as described [39] using the function lm() in R. We tested the significance of the selection coefficient, which is equal to the slope of the regression ß, by using a t-test to test the null hypothesis that ß = 0. 95% confidence intervals were computed in R using the standard error of the linear fit and the appropriate t-distribution depending on the degrees of freedom.

### Genotyping

We used quantitative sequencing to determine allele frequencies for all single nucleotide polymorphisms. Following confirmation of mutations identified using SNPScanner, we PCR amplified these genomic segments directly from the frozen glycerol stocks of heterogeneous populations. PCR products were cleaned up using Qiagen PCR cleanup kits and then sequenced in two different reactions using the forward and reverse primer. Control reactions using pure ancestral and evolved clones were also performed.

The relative allele frequency was determined from the resulting sequence traces using the program PeakPicker [59]. Peaks were normalized using the nearest six nucleotides of the same identity. Allele frequencies within population samples were determined by comparing the normalized value with the normalized value obtained from a homogeneous sample of the allele. 95% CI intervals were computed on the basis of independent sequencing reactions using a forward and reverse primer.

We developed TaqMan allelic discrimination assays for a subset of single nucleotide polymorphism alleles. Custom TaqMan SNP Genotyping Assays probe/primer sets consisting of an allele specific FAM and VIC probe and common primers were obtained from Applied Biosystems. Sequences for primer and probes are available upon request. In order to facilitate high-throughput analysis we developed a protocol for directly genotyping whole cells from frozen glycerol stocks. We diluted glycerol stocks 1∶5 in water and then added 2.75 µL to 2.50 µL of 2X TaqMan Universal PCR Mix (Applied Biosystems) and 0.25 µL of 20x Primer/Probe mix to a final volume of 5 µL. Otherwise, we followed standard procedures for allelic discrimination plate reads as suggested by the manufacturer (Applied Biosystems).

### Estimation of Fitness Coefficients during Evolution Experiments

We used allele frequency estimates from the evolution experiments to estimate the fitness coefficients for each mutation and each clone. For each mutation we used data from the first non-zero allele frequency until the allele frequency reached a maximum. This is not necessarily the last measured point as some alleles appeared to be stabilizing or even decreasing after initially increasing. By excluding those points our computed values are a maximal estimate of the fitness coefficient. Fitness coefficients were calculated by regressing ln(p/q) against the number of generations as described for the fitness assays above. We also computed a fitness coefficient for each clone by treating all allele frequency measurements at each time-point as independent measurements of a given clone's frequency.

### Fluctuation Test

Mutation rates were determined by fluctuation test [1]. 1000 cells were inoculated into 96-well culture plates and grown to saturation densities of ∼10^7^ cells/ml. 20 samples of each strain were plated to canavanine media. Mutation rates were calculated by the Poisson distribution from the proportion of plates with no canavanine resistant colonies.

### Statistical Methods

Pairwise pearson correlations were computed between the 16 clonal isolates within each selection. We excluded comparisons between the two clones derived from the same population and analyzed the resulting 112 comparisons. Probability density estimates were computed in R. In order to compare the three distributions we performed a Mann-Whitney rank-sum test.

## Supporting Information

Figure S1 Microarray analysis of predicted tranlocation PFGE bands.(A) Sample gel showing new bands in both clones from population G8. Chromosome ladder from wt strain shown at right. (B) Microarray analysis of all gel bands for predicted translocations (see Table S2). Enriched segments on the chromosomes of interest are shown in red. Segments not present in the translocation are colored gray. Complete data are available at GEO (Accession GSE13435).
(0.54 MB PDF)

Figure S2 Identification of a long terminal repeat (LTR) insertion in *SNF6*. (A) Analysis of tiling microarray data from clone G1c2 using the SNPScanner algorithm identified a predicted sequence variant in *SNF6*. PCR analysis identified a size polymorphism at this locus. Upon sequence confirmation we identified a 338bp insertion in *SNF6*. BLAST analysis identified the inserted sequence to be a single LTR derived from a Ty1 retrotransposon. (B) An LTR insertion is likely to be the result of a two-step process in which a full length retrotransposon insertion is followed by intrachromosomal recombination between tandem LTRs bounding the retrotransposon resulting in an orphan LTR. Curiously, the *SNF6* LTR insertion is bounded by an identical sequence motif of 5 bases (italicized and underlined sequence in figure). This motif is present once in the wildtype *SNF6* sequence suggesting that the second copy is either derived from the retrotransposon or has been duplicated during the insertion event.
(0.12 MB PDF)

Figure S3 CGH analysis of population samples to determine allele frequencies. We performed CGH on DNA samples derived from population samples harvested from the endpoint or near the endpoint of the evolutions. Copy number variants present at detectable frequencies in the population are indicated in red or green. Only population samples with detectable copy number changes are shown. Calculated frequencies: G6, 2 copies of chromosome 12 (24%); P3, 1 copy of chrIII segment (20%), 3 copies of chrV segment (47%); P5, 2 copies of chrVI segment (17%), 2 copies of chrXIII (17%); P6, 2 copies chrV segment (23%), 2 copies chrVI segment (23%); P7, 3 copies of chrIV (69%), 3 copies of chrVI (77%), 3 copies of chrX (75%), 3 copies of chrXVI (73%), 4 copies of chrXIII (66%).
(0.41 MB PDF)

Figure S4 Representative results of quantitative sequencing. We estimated allele frequencies by analyzing the electropherogram data using the program PeakPicker [59]. (A) Homogeneous DNA samples are used to identify the SNP of interest. At the polymorphic site, two peaks are reported in the electropherogram data. The relative height of these peaks, corresponding to the strength of the fluorescent signal is used to estimate the allele frequencies. (B) Application of quantitative sequencing to population trajectories enables estimation of allele frequencies across each evolution's history.
(3.36 MB PDF)

Figure S5 Representative results of Taqman allelic discrimination. We analyzed a subset of allele frequencies using Taqman allelic discrimination assays. Custom probes and primer sets were manufactured for each allele. 96 samples were analyzed in quadruplicate using an ABI 9700T plate reader. In each plate we included a no template control (green asterisk), allele control (pink triangle) and evolved allele control (gray plus signs). We used a custom k-medians clustering algorithm to assign genotypes to ancestral (blue circles) or evolved (red circles) state.
(0.10 MB PDF)

Figure S6 Comparison of SNP allele frequency estimations using quantitative sequencing and Taqman allelic discrimination. We compared allele frequency estimates using quantitative sequencing (red) with those obtained by genotyping clonal isolates using TaqMan allelic discrimination analysis (green) for two alleles: (A) *CCR4* E724V and (chrI:111188T->A) (B) *SAP185* synonymous (chrX: 243203G->A). 95% confidence intervals are shown. We found high concordance between the allele frequency profiles generated using both methods thereby validating the use of quantitative sequencing, which is more amenable to high throughput analysis as it can be performed on population samples rather than requiring analysis of individuals.
(0.28 MB PDF)

Figure S7 Clonal competition assays reveal fitness benefit per culture generation. We competed clonal isolates against the ancestral strain as described (see Methods). In order to determine the growth advantage per generation we fit the data by modeling ln(p/q) = s * (generations) using ordinary least squares. (A) The clonal isolate G1c1 contains 9 confirmed nucleotide variants but no transporter amplifications. In a competition experiment in a glucose limited chemostat it out-competes the ancestral strain with a relative selective advantage, s = 1.106±0.012. (B) Multiple copies of *SUL1* confer a strong selective advantage in sulfate limiting conditions. We obtained the *SUL1* gene on a high copy plasmid and competed the resulting strain against the wildtype ancestor. Multiple copies of *SUL1* confer a 50% growth advantage per generation (s = 1.49±0.093). (C) The drug resistance marker, Can^R^, confers a negligible fitness contribution under these conditions. By competing a drug resistant version of the ancestral strain with a wild type version we detected a slight fitness advantage in the drug resistant strain of approximately 1% per generation (s = 1.015±0.001).
(0.25 MB PDF)

Figure S8 Meiotic separation of alleles identifies advantageous allele. Following a backcross of evolved clone P1c2 to the ancestral strain meiotic segregants were isolated and competed against one another in a chemostat. We performed two experiments: (A) one in which only the MATa segregants were included and (B) one in which only MATα segregants were included. In both experiments the *CKA2* allele quickly swept to fixation (blue diamonds) while the *SIR1* allele (green circles) remained around the neutral frequency of 50%.
(0.22 MB PDF)

Table S1 Complete GO term enrichment results for clusters 1-13 summarized in Figure 1A. In addition we performed GO term enrichment on the set of 689 genes that did not change in expression during evolutions.
(0.21 MB XLS)

Table S2 Summary of structural variation in genomes of evolved clones.
(0.09 MB DOC)

Table S3 Segregation analysis of *SUL1* amplification using CGH.
(0.04 MB DOC)

Table S4 Transposon specific extraction results.
(0.04 MB DOC)

Table S5 Processed gene expression microarray data for all clones and populations.
(7.69 MB XLS)

Table S6 Processed CGH data for all clones and populations.
(4.12 MB XLS)
