# Supplementary figures and images for: The Repertoire and Dynamics of Evolutionary Adaptations to Controlled Nutrient-Limited Environments in Yeast
Source: PLoS Genet. 2008 Dec 12;4(12):e1000303. doi: 10.1371/journal.pgen.1000303 (PMC2586090; doi:10.1371/journal.pgen.1000303)

A.

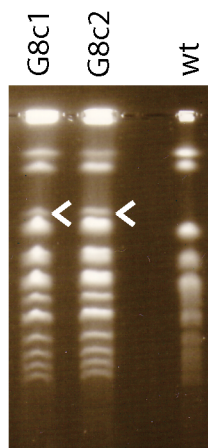

bands excised  
and hybridized  
to microarray

S3c1

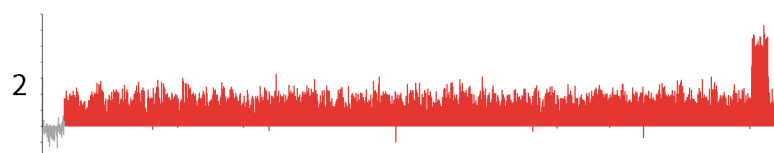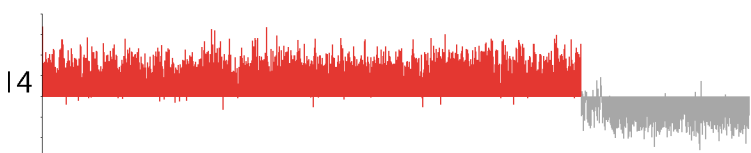

P6c1

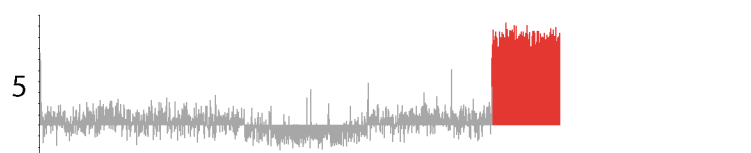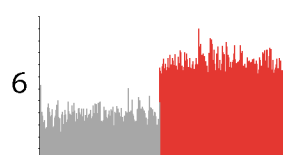

G7c1

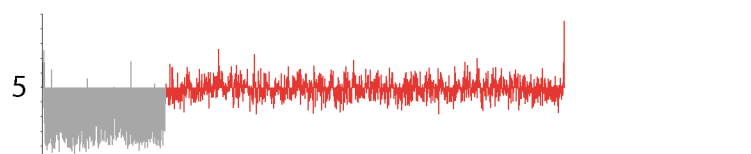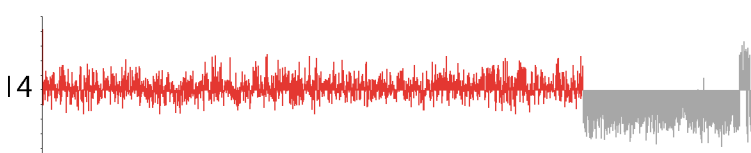

G8c1

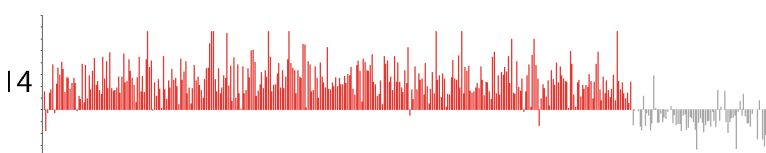

B.

S8c2

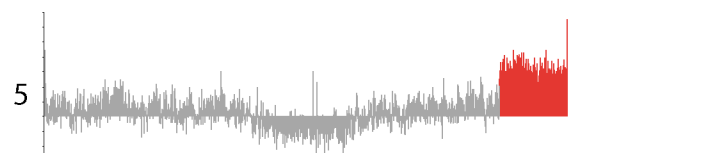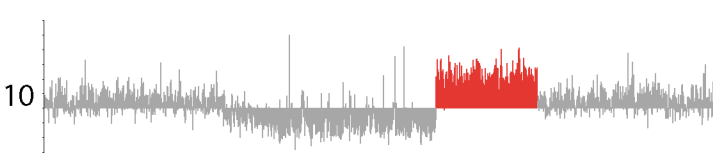

S3c2

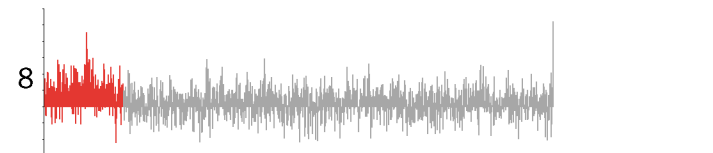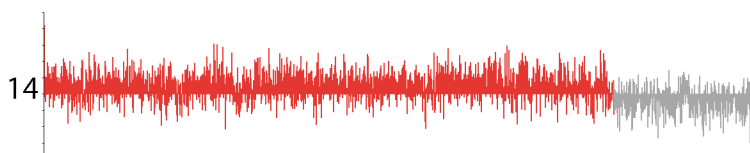

P6c2

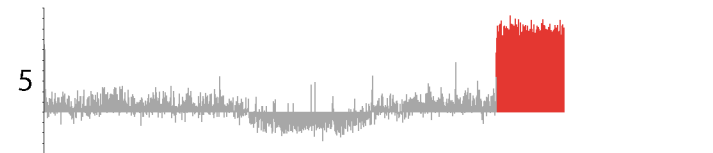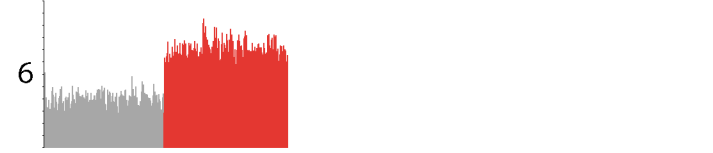

G7c2

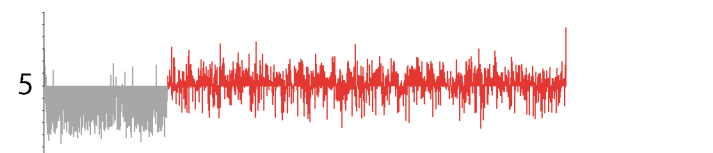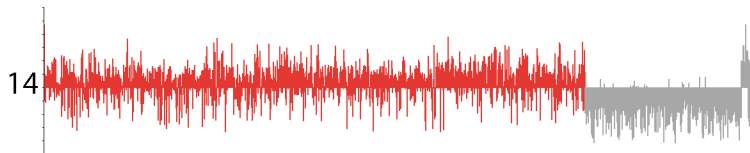

G8c2

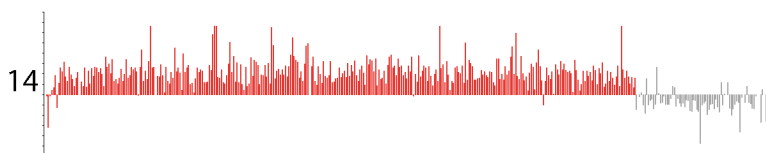

Supplement: Figure S1 — Microarray analysis of predicted tranlocation PFGE bands.(A) Sample gel showing new bands in both clones from population G8. Chromosome ladder from wt strain shown at right. (B) Microarray analysis of all gel bands for predicted translocations (see Table S2). Enriched segments on the chromosomes of interest are shown in red. Segments not present in the translocation are colored gray. Complete data are available at GEO (Accession GSE13435). (0.54 MB PDF) [file pgen.1000303.s001.pdf]

A

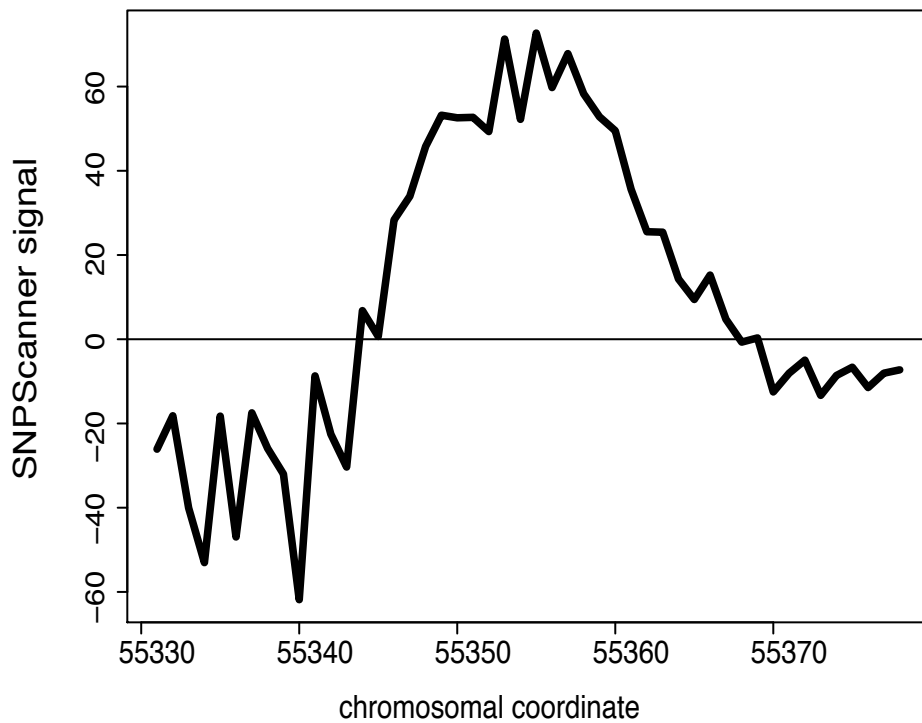

B

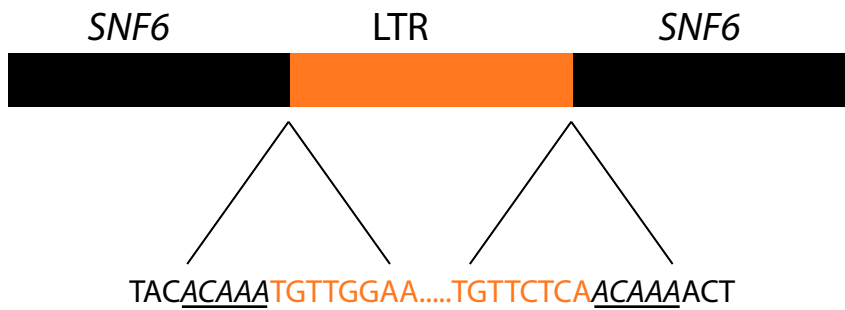

Supplement: Figure S2 — Identification of a long terminal repeat (LTR) insertion in SNF6. (A) Analysis of tiling microarray data from clone G1c2 using the SNPScanner algorithm identified a predicted sequence variant in SNF6. PCR analysis identified a size polymorphism at this locus. Upon sequence confirmation we identified a 338bp insertion in SNF6. BLAST analysis identified the inserted sequence to be a single LTR derived from a Ty1 retrotransposon. (B) An LTR insertion is likely to be the result of a two-step process in which a full length retrotransposon insertion is followed by intrachromosomal recombination between tandem LTRs bounding the retrotransposon resulting in an orphan LTR. Curiously, the SNF6 LTR insertion is bounded by an identical sequence motif of 5 bases (italicized and underlined sequence in figure). This motif is present once in the wildtype SNF6 sequence suggesting that the second copy is either derived from the retrotransposon or has been duplicated during the insertion event. (0.12 MB PDF) [file pgen.1000303.s002.pdf]

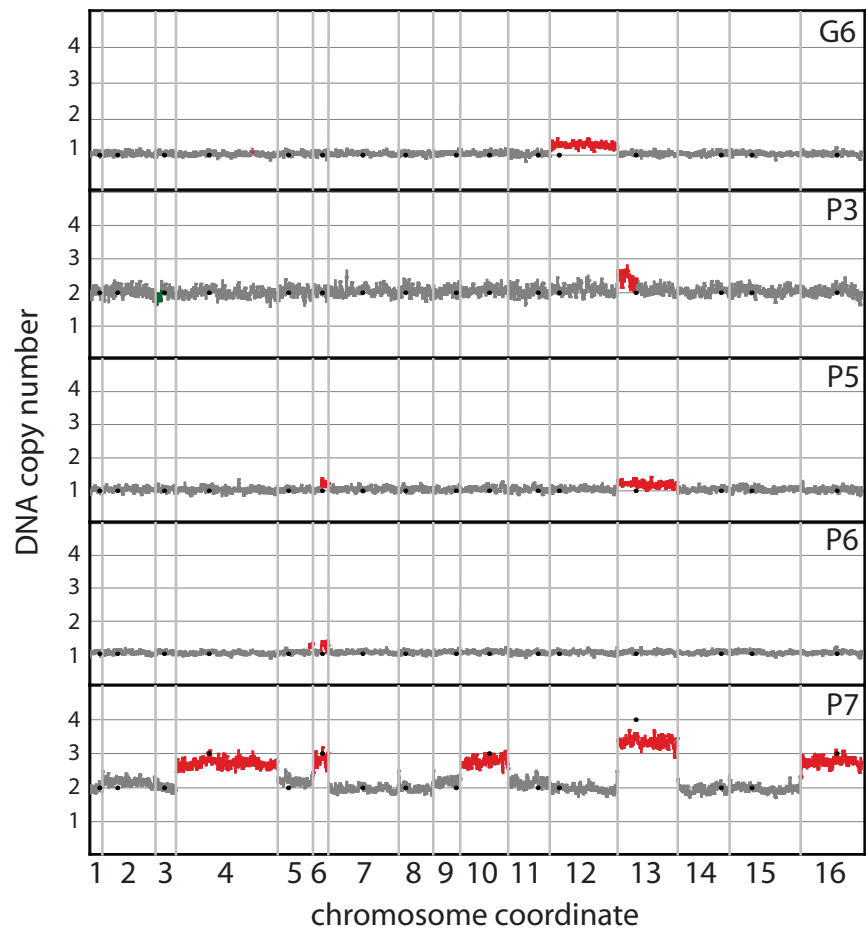

Supplement: Figure S3 — CGH analysis of population samples to determine allele frequencies. We performed CGH on DNA samples derived from population samples harvested from the endpoint or near the endpoint of the evolutions. Copy number variants present at detectable frequencies in the population are indicated in red or green. Only population samples with detectable copy number changes are shown. Calculated frequencies: G6, 2 copies of chromosome 12 (24%); P3, 1 copy of chrIII segment (20%), 3 copies of chrV segment (47%); P5, 2 copies of chrVI segment (17%), 2 copies of chrXIII (17%); P6, 2 copies chrV segment (23%), 2 copies chrVI segment (23%); P7, 3 copies of chrIV (69%), 3 copies of chrVI (77%), 3 copies of chrX (75%), 3 copies of chrXVI (73%), 4 copies of chrXIII (66%). (0.41 MB PDF) [file pgen.1000303.s003.pdf]

A

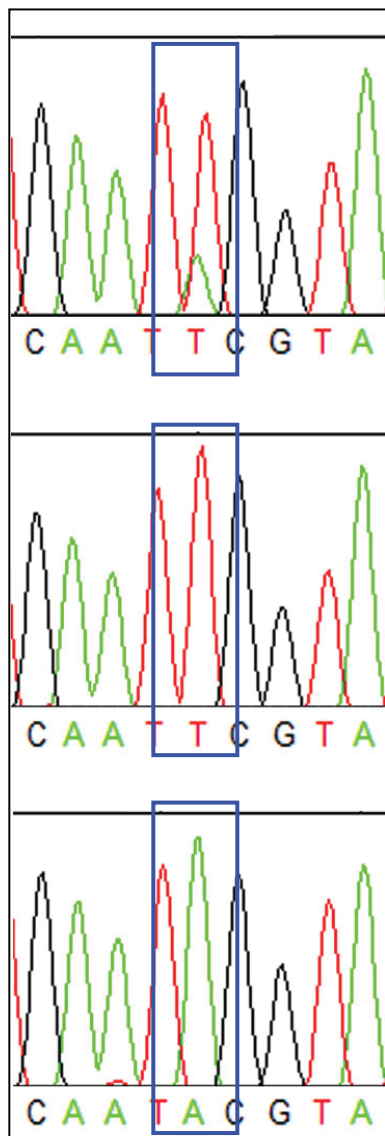

B

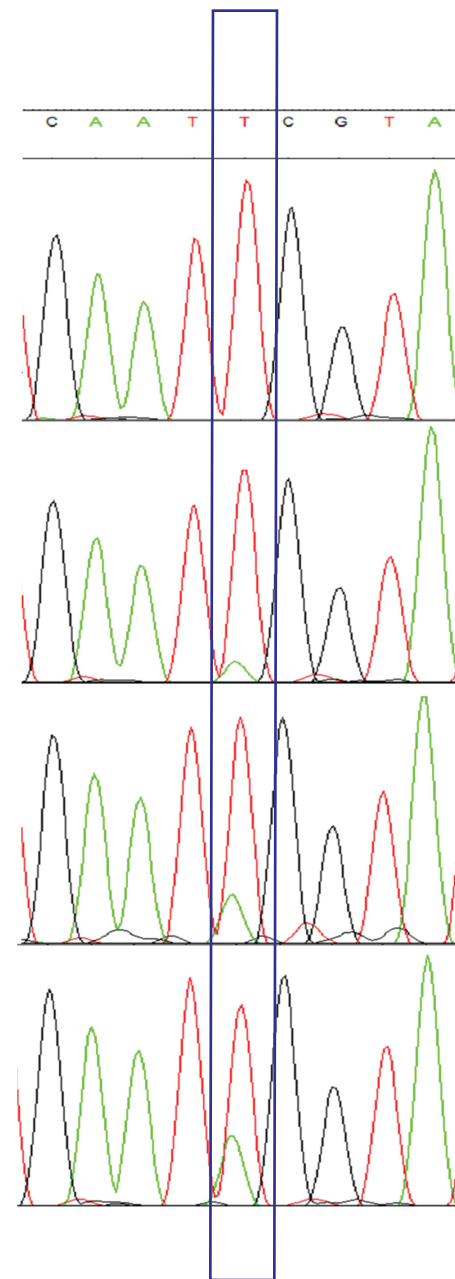

Supplement: Figure S4 — Representative results of quantitative sequencing. We estimated allele frequencies by analyzing the electropherogram data using the program PeakPicker [59]. (A) Homogeneous DNA samples are used to identify the SNP of interest. At the polymorphic site, two peaks are reported in the electropherogram data. The relative height of these peaks, corresponding to the strength of the fluorescent signal is used to estimate the allele frequencies. (B) Application of quantitative sequencing to population trajectories enables estimation of allele frequencies across each evolution's history. (3.36 MB PDF) [file pgen.1000303.s004.pdf]

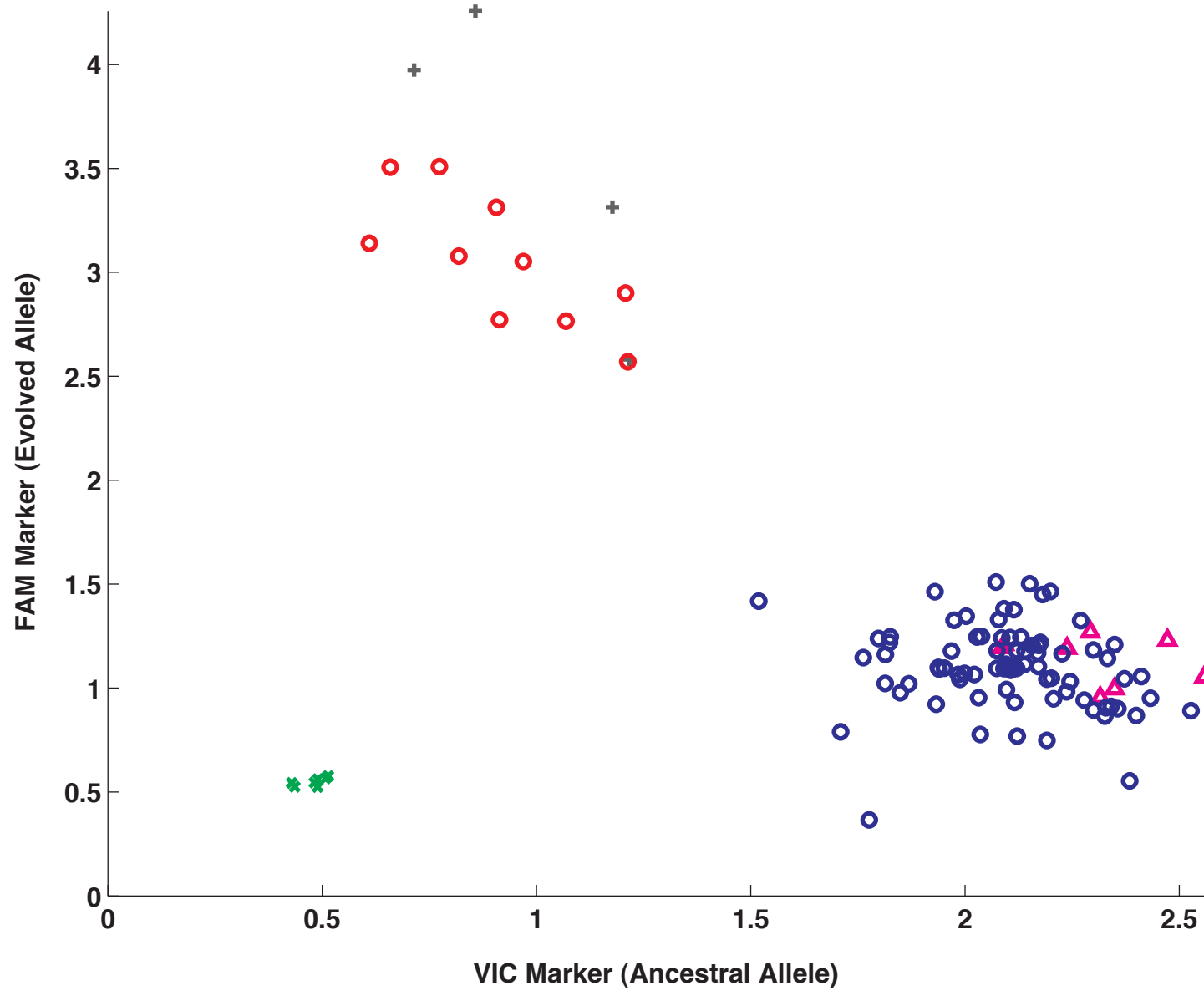

Supplement: Figure S5 — Representative results of Taqman allelic discrimination. We analyzed a subset of allele frequencies using Taqman allelic discrimination assays. Custom probes and primer sets were manufactured for each allele. 96 samples were analyzed in quadruplicate using an ABI 9700T plate reader. In each plate we included a no template control (green asterisk), allele control (pink triangle) and evolved allele control (gray plus signs). We used a custom k-medians clustering algorithm to assign genotypes to ancestral (blue circles) or evolved (red circles) state. (0.10 MB PDF) [file pgen.1000303.s005.pdf]

A

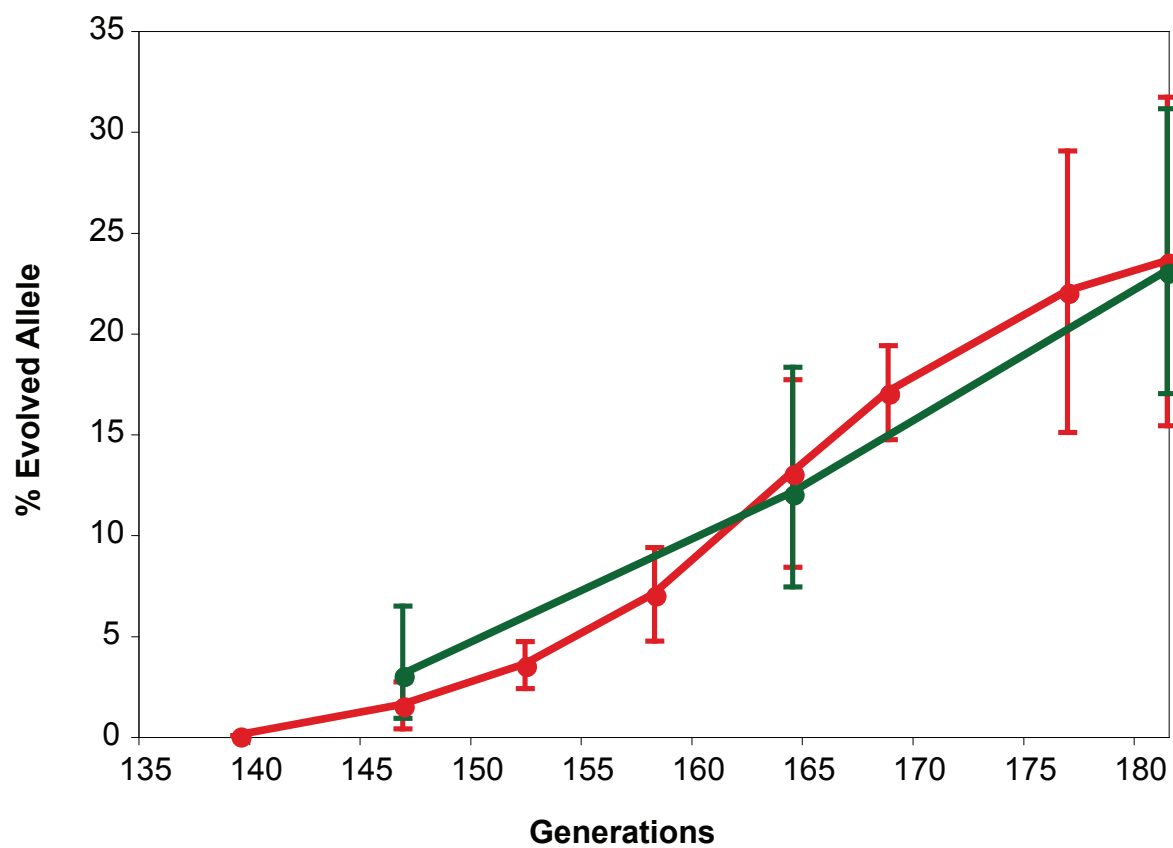

B

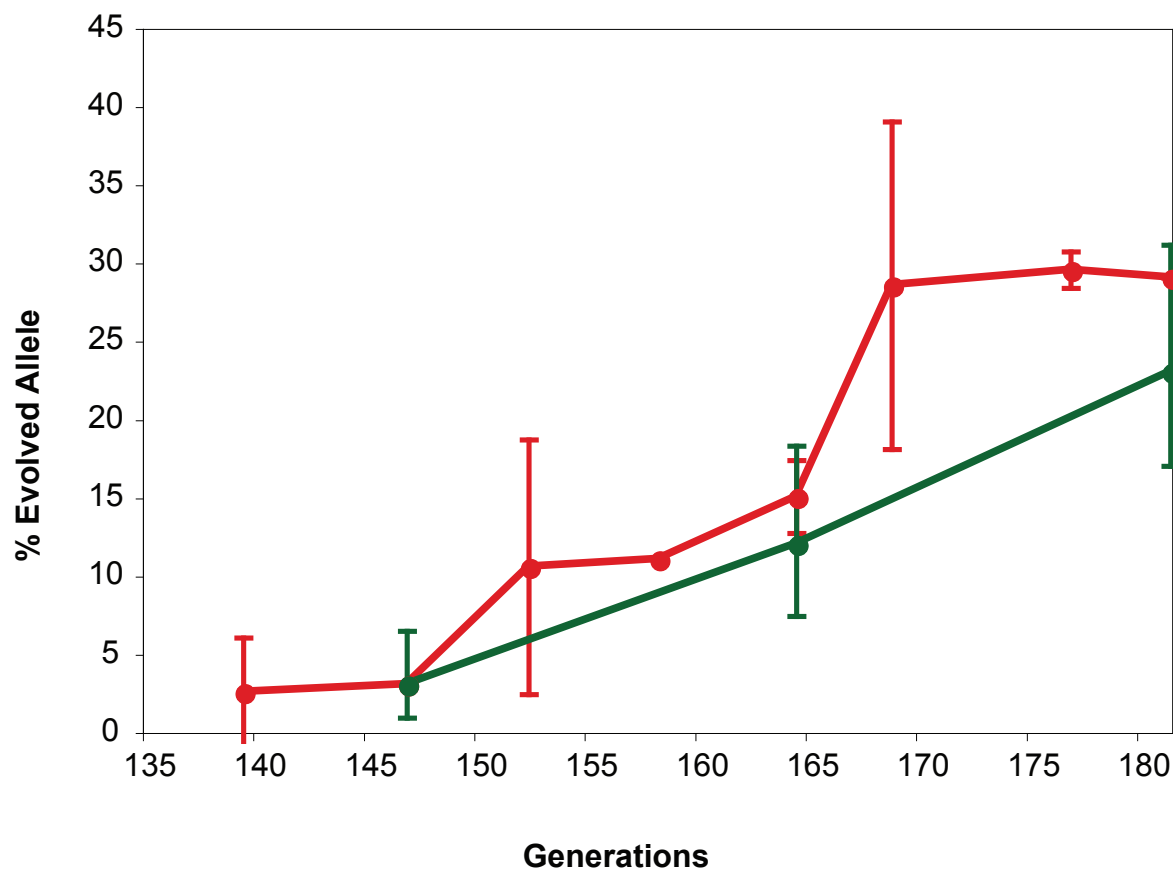

Supplement: Figure S6 — Comparison of SNP allele frequency estimations using quantitative sequencing and Taqman allelic discrimination. We compared allele frequency estimates using quantitative sequencing (red) with those obtained by genotyping clonal isolates using TaqMan allelic discrimination analysis (green) for two alleles: (A) CCR4 E724V and (chrI:111188T->A) (B) SAP185 synonymous (chrX: 243203G->A). 95% confidence intervals are shown. We found high concordance between the allele frequency profiles generated using both methods thereby validating the use of quantitative sequencing, which is more amenable to high throughput analysis as it can be performed on population samples rather than requiring analysis of individuals. (0.28 MB PDF) [file pgen.1000303.s006.pdf]

A

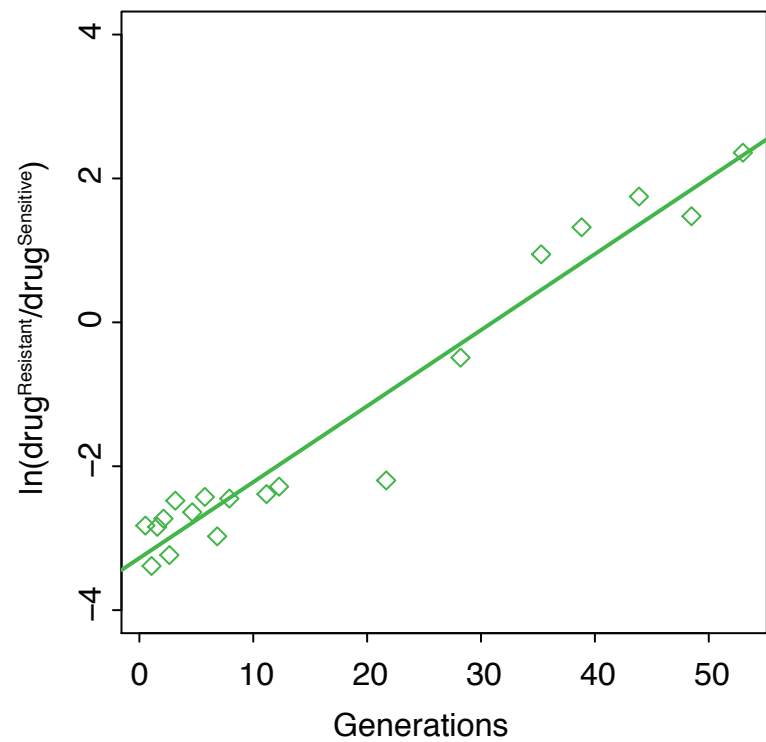

B

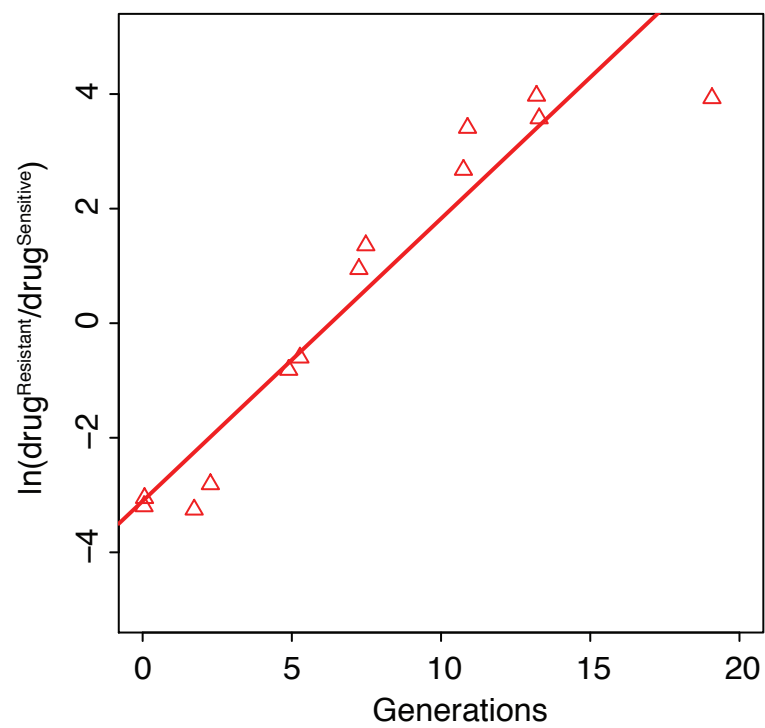

C

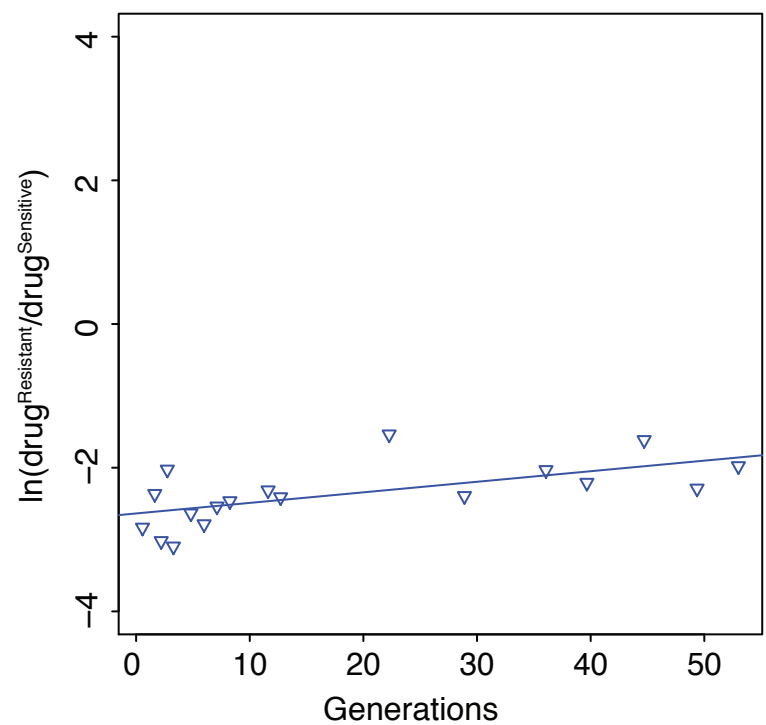

Supplement: Figure S7 — Clonal competition assays reveal fitness benefit per culture generation. We competed clonal isolates against the ancestral strain as described (see Methods). In order to determine the growth advantage per generation we fit the data by modeling ln(p/q) = s * (generations) using ordinary least squares. (A) The clonal isolate G1c1 contains 9 confirmed nucleotide variants but no transporter amplifications. In a competition experiment in a glucose limited chemostat it out-competes the ancestral strain with a relative selective advantage, s = 1.106±0.012. (B) Multiple copies of SUL1 confer a strong selective advantage in sulfate limiting conditions. We obtained the SUL1 gene on a high copy plasmid and competed the resulting strain against the wildtype ancestor. Multiple copies of SUL1 confer a 50% growth advantage per generation (s = 1.49±0.093). (C) The drug resistance marker, CanR, confers a negligible fitness contribution under these conditions. By competing a drug resistant version of the ancestral strain with a wild type version we detected a slight fitness advantage in the drug resistant strain of approximately 1% per generation (s = 1.015±0.001). (0.25 MB PDF) [file pgen.1000303.s007.pdf]

A

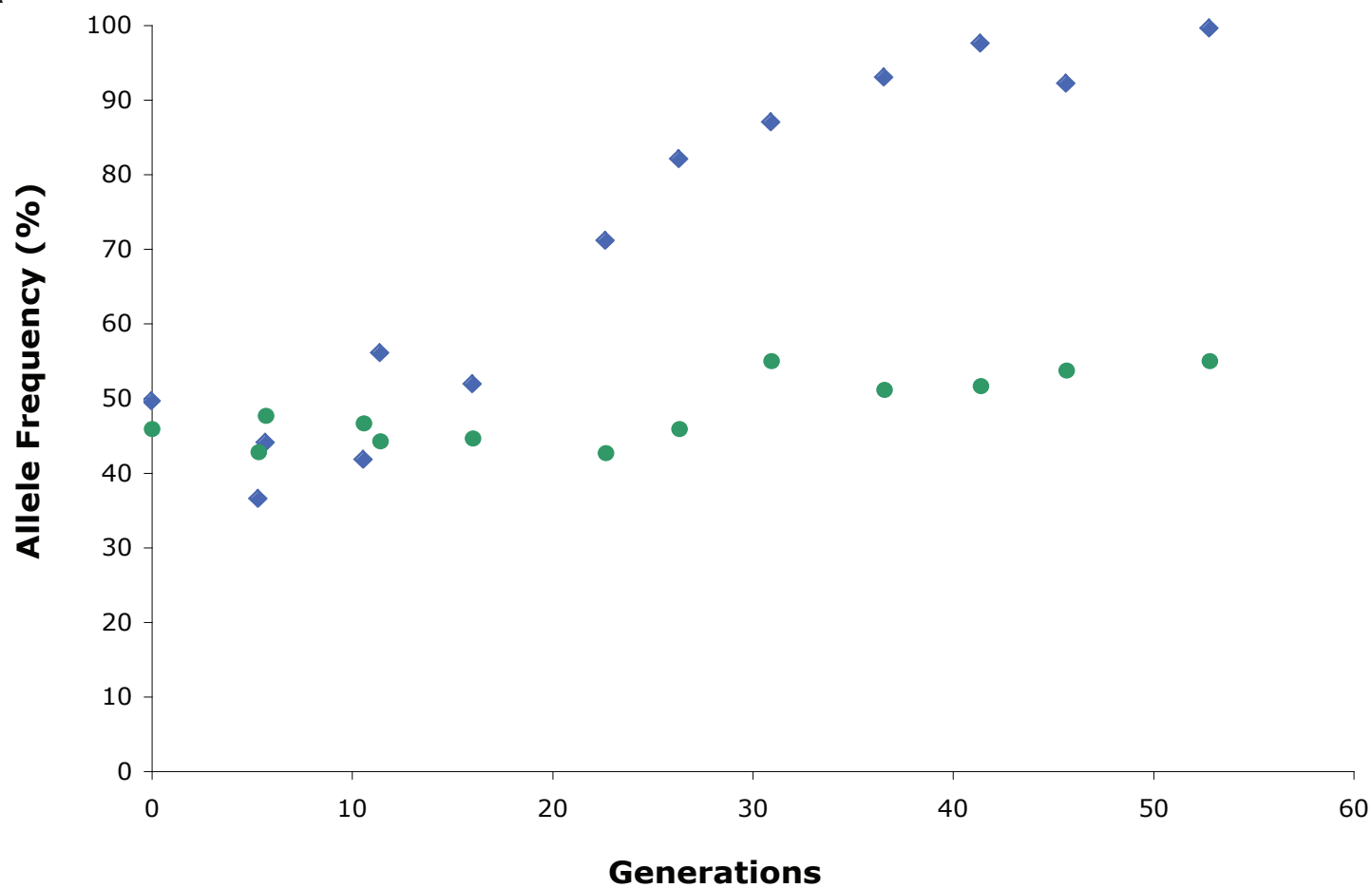

B

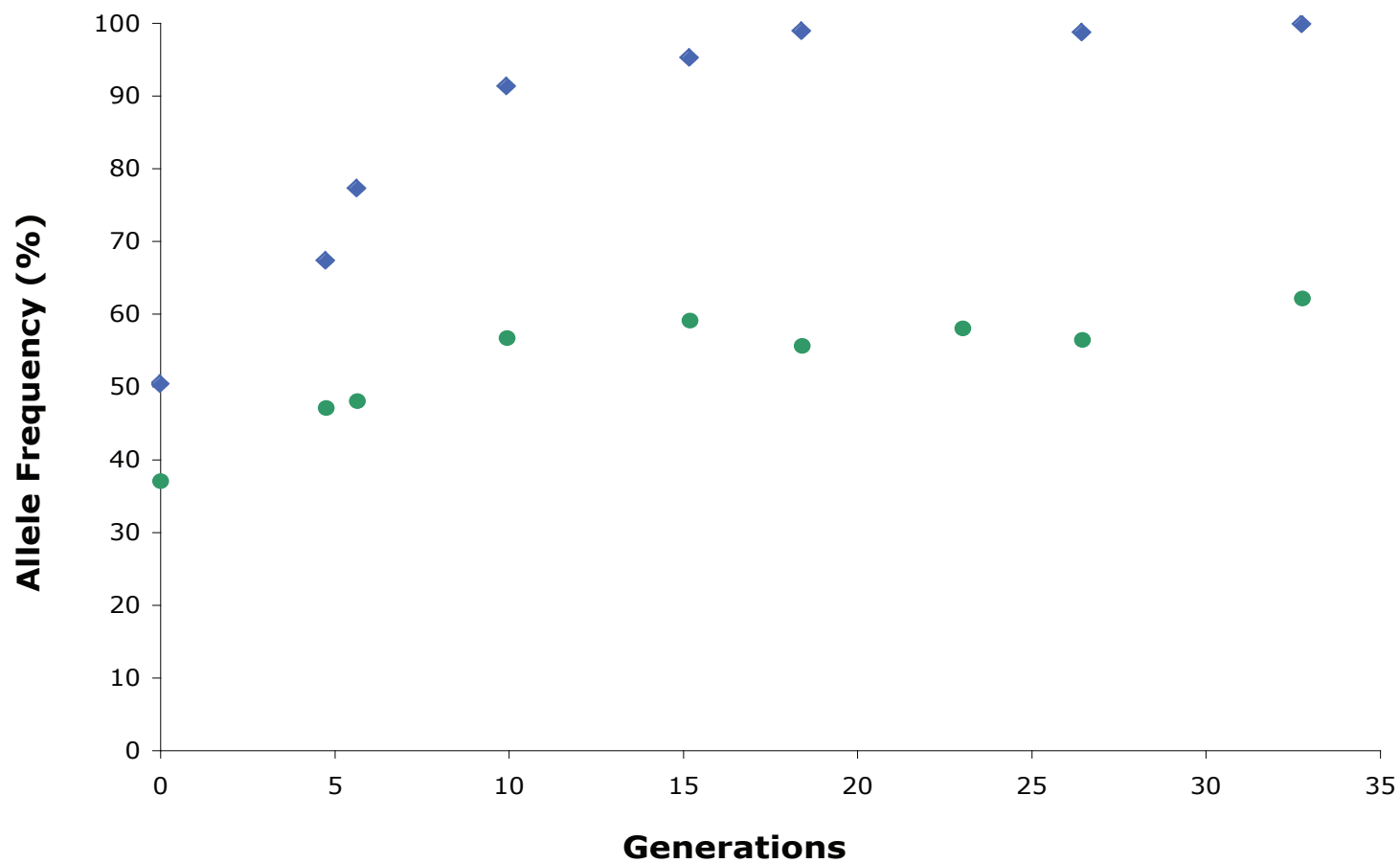

Supplement: Figure S8 — Meiotic separation of alleles identifies advantageous allele. Following a backcross of evolved clone P1c2 to the ancestral strain meiotic segregants were isolated and competed against one another in a chemostat. We performed two experiments: (A) one in which only the MATa segregants were included and (B) one in which only MATα segregants were included. In both experiments the CKA2 allele quickly swept to fixation (blue diamonds) while the SIR1 allele (green circles) remained around the neutral frequency of 50%. (0.22 MB PDF) [file pgen.1000303.s008.pdf]
