# Supplementary material for: The Repertoire and Dynamics of Evolutionary Adaptations to Controlled Nutrient-Limited Environments in Yeast
Source: PLoS Genet. 2008 Dec 12;4(12):e1000303. doi: 10.1371/journal.pgen.1000303 (PMC2586090; doi:10.1371/journal.pgen.1000303)
Supplement: Table S2 — Summary of structural variation in genomes of evolved clones. (0.09 MB DOC) [file pgen.1000303.s010.doc]

**Table S2. Structural variation identified in the genomes of evolved clones.** Gross chromosomal events were assayed using a combination of array CGH and pulse field gel electrophoresis. Repeated events identified in multiple clones are highlighted in the same color.

| **Evolved Clone** | **Strain Back-ground** | **Ploidy** | **Copy number change** **in nutrient-specific gene** | **Gross chromosomal rearrangements and extra chromosomes** | **Repetitive sequences at breakpoint** |
| --- | --- | --- | --- | --- | --- |
| P3c1 | S288c | 2N |  | chr12 size change |  |
| P3c2 | S288c | 2N |  | chr12 size change |  |
| P4c1 | S288c | 2N |  | chr12 size change |  |
| P5c1 | CEN.PK | 1N | *PHO5* deletion |  |  |
| P5c2 | CEN.PK | 1N | *PHO5* deletion |  |  |
| P6c1 | CEN.PK | 1N |  | translocation chr5[*YER161C*-*TEL05R*] chr6[*YFL002C*-*TEL06R*] resulting in 2 copies of both segments | tRNA and Ty1 tRNA, Ty2, tau, delta |
| P6c2 | CEN.PK | 1N |  | translocation chr5[*YER161C*-*TEL05R*] chr6[*YFL002C*-*TEL06R*] resulting in 2 copies of both segments | tRNA and Ty1 tRNA, Ty2, tau, delta |
| P7c1 | CEN.PK | 2N | *PHO5* amplification | 3 copies of chrs 4, 6, 10, and 16 4 copies of chr13 |  |
| P7c2 | CEN.PK | 2N | *PHO5* amplification | 3 copies of chrs 4, 6, 10, and 16 4 copies of chr13 |  |
| S1c2 | S288c | 1N | *SUL1* amplification |  |  |
| S2c1 | S288c | 1N | *SUL1* amplification |  |  |
| S2c2 | S288c | 1N | *SUL1* amplification |  |  |
| S3c1 | S288c | 2N | *SUL1* amplification | translocation chr2[*YBL100C*-*TEL04R*] chr14[*TEL14L*-*YNL017C*] resulting in 1 copy of chr2[*TEL04L*-*YBL101C*] chr14[*YNL016W*-*TEL14R*] | Ty2, delta, tau, tRNA tRNA |
| S3c2 | S288c | 2N | *SUL1* amplification | chr12 size change translocation chr8[*TEL08L*-*YHL009C*] chr14[*TEL14L*-*YNR001W-A*] resulting in 3 copies of chr8 segment and 1 copy of chr14[*YNR002C*-*TEL14R*] | tRNA, sigma, Ty4, deltas tRNAs, delta, tau |
| S4c1 | S288c | 2N | *SUL1* amplification |  |  |
| S4c2 | S288c | 2N | *SUL1* amplification |  |  |
| S5c1 | CEN.PK | 1N | *SUL1* amplification |  |  |
| S5c2 | CEN.PK | 1N | *SUL1* amplification |  |  |
| S6c1 | CEN.PK | 1N | *SUL1* amplification |  |  |
| S6c2 | CEN.PK | 1N | *SUL1* amplification |  |  |
| S7c1 | CEN.PK | 2N | *SUL1* amplification |  |  |
| S7c2 | CEN.PK | 2N | *SUL1* amplification |  |  |
| S8c1 | CEN.PK | 2N | *SUL1* amplification |  |  |
| S8c2 | CEN.PK | 2N | *SUL1* amplification | translocation chr5[*YER161C*-*TEL05R*] chr10[*YJL005W*-*YJR055W*] resulting in 4 copies of chr5 segment and 3 copies of chr10 segment | tRNA and Ty1 L: tau, delta, tRNAs R: deltas and tRNAs |
| G2c1 | S288c | 1N | *HXT6/7* amplification |  |  |
| G5c1 | CEN.PK | 1N | *HXT6/7* amplification |  |  |
| G5c2 | CEN.PK | 1N | *HXT6/7* amplification |  |  |
| G6c1 | CEN.PK | 1N | *HXT6/7* amplification |  |  |
| G6c2 | CEN.PK | 1N | *HXT6/7* amplification |  |  |
| G7c1 | CEN.PK | 2N | *HXT6/7* amplification | 3 copies chr1 translocation chr5[*YEL010W*-*TEL05R*] chr14[*TEL14L*-*YNL017C*] resulting in 3 copies of chr5 segment and 1 copy of chr14[*YNR002C*-*TEL14R*] | tRNA, delta, new Ty insertion tRNA |
| G7c2 | CEN.PK | 2N | *HXT6/7* amplification | chr12 size change translocation chr5[*YEL010W*-*TEL05R*] chr14[*TEL14L*-*YNL017C*] resulting in 3 copies of chr5 segment and 1 copy of chr14[*YNR002C*-*TEL14R*] | tRNA, delta, new Ty insertion tRNA |
| G8c1 | CEN.PK | 2N | *HXT6/7* amplification | translocation chr14[*TEL14L*-*YNL036W*] chr14[*TEL14L*-*YNR001W-A*] resulting in 3 copies of the chr14L fragment and 1 copy of chr14[*YNR002C*-*TEL14R*] | tRNAs, tau, Ty2 tRNAs, delta, tau |
| G8c2 | CEN.PK | 2N | *HXT6/7* amplification | 3 copies chr3 translocation chr14[*TEL14L*-*YNL036W*] chr14[*TEL14L*-*YNR001W-A*] resulting in 3 copies of the chr14L fragment and 1 copy of chr14[*YNR002C*-*TEL14R*] | tRNAs, tau, Ty2 tRNAs, delta, tau |
