# Supplementary material for: The Repertoire and Dynamics of Evolutionary Adaptations to Controlled Nutrient-Limited Environments in Yeast
Source: PLoS Genet. 2008 Dec 12;4(12):e1000303. doi: 10.1371/journal.pgen.1000303 (PMC2586090; doi:10.1371/journal.pgen.1000303)
Supplement: Table S3 — Segregation analysis of SUL1 amplification using CGH. (0.04 MB DOC) [file pgen.1000303.s011.doc]

**Table S3. Segregation analysis of *SUL1* amplification using aCGH.** We subjected the four meiotic products from the sporulation of diploid clones S4c1 and S4c2 as well as a backcross of the haploid clone S5c1 to aCGH analysis. The average *SUL1* copy number was determined for each segregant using 11 probes spanning the gene. Increased SUL1 copy number segregated as a mendelian locus consistent with a tandem repeat.

| **Clone** | **Segregant** | **Average copy number of *SUL1*** | ***SUL1* allele** |
| --- | --- | --- | --- |
| S4c1 | A | 1.1 ± 0.1 | wt |
|  | B | 3.2 ± 0.4 | amplified |
|  | C | 1.0 ± 0.1 | wt |
|  | D | 3.2 ± 0.3 | amplified |
| S4c2 | A | 5.3 ± 0.5 | amplified |
|  | B | 5.6 ± 0.5 | amplified |
|  | C | 1.1 ± 0.1 | wt |
|  | D | 1.1 ± 0.1 | wt |
| S5c1 x ancestor | A | 0.9 ± 0.1 | wt |
|  | B | 4.8 ± 0.3 | amplified |
|  | C | 0.9 ± 0.1 | wt |
|  | D | 1.9 ± 0.2 | amplified |
