# Supplementary material for: The Repertoire and Dynamics of Evolutionary Adaptations to Controlled Nutrient-Limited Environments in Yeast
Source: PLoS Genet. 2008 Dec 12;4(12):e1000303. doi: 10.1371/journal.pgen.1000303 (PMC2586090; doi:10.1371/journal.pgen.1000303)
Supplement: Table S4 — Transposon specific extraction results. (0.04 MB DOC) [file pgen.1000303.s012.doc]

**Table S4. New Ty1 and Ty2 insertions.** One clone from each population was analyzed by Transposon Specific Extraction and compared with the matching ancestral strain. Only clones with novel insertions are listed.

| **Clone** | **Ty location** | **Confirmation** | **Nearby repetitive sequences** |
| --- | --- | --- | --- |
| P2c2 | intergenic region between *PDH1* and *YPR003C* | Tiling array | tRNA  delta element |
| P6c1 | Intergenic region between *SEN34* and *BUD14* | Tiling array | *YARCTy1-1**  tRNA |
| P8c1 | Intergenic region between *SEN34* and *BUD14* | - | *YARCTy1-1**  tRNA |
| G2c2 | MTH1 | Tiling array and PCR | tRNA |
| G6c1 | NUT1 | Tiling array |  |
| G7c1 | Intergenic region between GLC3 and GCN4^†^ | - | 2 tRNAs  3 delta elements |

*Ty1 found in wt S288c but not present in wt CEN.PK strains.

^†^Region also used as a GCR breakpoint in this strain.
